# The Chromatin Remodeler CHD8 Is Required for Activation of Progesterone Receptor-Dependent Enhancers

**DOI:** 10.1371/journal.pgen.1005174

**Published:** 2015-04-20

**Authors:** María Ceballos-Chávez, Alicia Subtil-Rodríguez, Eugenia G. Giannopoulou, Daniel Soronellas, Elena Vázquez-Chávez, Guillermo P. Vicent, Olivier Elemento, Miguel Beato, José C. Reyes

**Affiliations:** 1 Molecular Biology Department, Centro Andaluz de Biología Molecular y Medicina Regenerativa (CABIMER), Consejo Superior de Investigaciones Científicas (CSIC), Seville, Spain; 2 Biological Sciences Department, New York City College of Technology, City University of New York, Brooklyn, New York, New York, United States of America; 3 Arthritis and Tissue Degeneration Program and the David Z. Rosensweig Genomics Research Center, Hospital for Special Surgery, New York, New York, United States of America; 4 Centre for Genomic Regulation (CRG), Barcelona, Spain; 5 Universitat Pompeu Fabra (UPF), Barcelona, Spain; 6 HRH Prince Alwaleed Bin Talal Bin Abdulaziz Alsaud Institute for Computational Biomedicine, Weill Cornell Medical College, Cornell University, New York, New York, United States of America; Case Western Reserve University, UNITED STATES

## Abstract

While the importance of gene enhancers in transcriptional regulation is well established, the mechanisms and the protein factors that determine enhancers activity have only recently begun to be unravelled. Recent studies have shown that progesterone receptor (PR) binds regions that display typical features of gene enhancers. Here, we show by ChIP-seq experiments that the chromatin remodeler CHD8 mostly binds promoters under proliferation conditions. However, upon progestin stimulation, CHD8 re-localizes to PR enhancers also enriched in p300 and H3K4me1. Consistently, CHD8 depletion severely impairs progestin-dependent gene regulation. CHD8 binding is PR-dependent but independent of the pioneering factor FOXA1. The SWI/SNF chromatin-remodelling complex is required for PR-dependent gene activation. Interestingly, we show that CHD8 interacts with the SWI/SNF complex and that depletion of BRG1 and BRM, the ATPases of SWI/SNF complex, impairs CHD8 recruitment. We also show that CHD8 is not required for H3K27 acetylation, but contributes to increase accessibility of the enhancer to DNaseI. Furthermore, CHD8 was required for RNAPII recruiting to the enhancers and for transcription of enhancer-derived RNAs (eRNAs). Taken together our data demonstrate that CHD8 is involved in late stages of PR enhancers activation.

## Author Summary

A lot of research has been devoted during the last decades to understand the mechanisms that control gene promoters activity, however, much less is known about enhancers. Only recently, the use of genome-wide chromatin immunoprecipitation techniques has revealed the existence of more than 400,000 enhancers in the human genome. We are starting to understand the importance of these regulatory elements and how they are activated or repressed. In this work we discover that the chromatin remodeler CHD8 is recruited to Progesteron Receptor-dependent enhancers upon hormone treatment. CHD8 is required for late steps in the activation of these enhancers, including transcription of the enhancers and synthesis of eRNA (long noncoding RNAs derived form the enhancers).

## Introduction

During the last decade it has become clear that regulation of gene transcription is accompanied by extensive changes in the chromatin organization of promoters [1]. More recently, efforts have concentrated on elucidating the chromatin dynamics of distal regulatory regions and particularly enhancers [2,3,4]. Enhancers were originally defined as regulatory sequences that can activate gene expression independently of their proximity or orientation with respect to their target genes [5]. Even though histone modifications signatures (e.g., high levels of histone H3 lysine 4 monomethyl (H3K4me1) and H3K27 acetyl (H3K27ac) modifications) have provided insight for the discovery of enhancer-like regions, the mechanisms and the factors that control enhancers activation are not yet well known.

Chromatin changes are normally performed by two types of enzymes: enzymes that chemically modify histones or ATP-dependent chromatin remodelers. ATP-dependent remodelling is performed by enzymes of the SNF2 family that use the energy of ATP hydrolysis to destabilize the interaction between DNA and histones [6,7]. In humans there are 26 ATPases of this family with specific roles in gene transcription and in other aspects of DNA metabolism. One of these ATPases is CHD8 which, in addition to the ATPase domain, contains two chromodomains in the amino terminus of the protein, and two BRK domains in the carboxy terminus [8]. CHD8 is able to remodel nucleosomes *in vitro* in an ATP-dependent reaction [9]; however, its *in vivo* functions are unclear. Inactivation of CHD8 by homologous recombination in mice provokes a strong growth retardation from embryonic day 5.5 and developmental arrest accompanied by massive apoptosis [10]. Ishihara *et al*. reported that CHD8 interacts with CTCF and plays a role in insulation activity [11]. It has also been reported that CHD8 represses beta-catenin target genes, and suppresses p53-dependent activation and apoptosis, by promoting histone H1 recruitment [9,12,13,14]. A role in repression of MLH1 gene, associated to MAFG, has been also shown [15]. In contrast to this repressive role, we have shown that CHD8 is required for E2F-dependent activation of G1/S specific promoters [16,17]. Additionally, it has been reported that CHD8 is required for estrogen-dependent induction of Cyclin E2 gene [18], and for recruitment of androgen receptor (AR) and activation of the *TMPRSS2* gene [19]. These two studies indicate that CHD8 is also involved in steroid hormone-dependent transcriptional regulation although the mechanisms of this regulation are unknown.

In this work, we have investigated the role of CHD8 in progesterone-dependent transcriptional regulation. Progesterone controls transcription through a complex mechanism [20]. On the one hand, progesterone-bound progesterone receptor (PR) is able to bind specific DNA sequences in chromatin, called PRE, and to recruit histone modification enzymes and ATP-dependent chromatin remodelers, such as NURF and BAF complexes (a member of the SWI/SNF complex family) [21,22]. On the other hand, a small fraction of PR is attached to the cytoplasmic side of the cell membrane and, in the presence of hormone, interacts with tyrosine kinases provoking the activation of various kinase cascades, including ERK1/ERK2 [23,24]. Activated ERK1/ERK2 phosphorylates PR and the kinase MSK1, forming a ternary complex that binds to chromatin. Recent genome-wide studies demonstrated that most PR binding sites (PRbs) are distal regulatory regions that map at introns and intergenic regions and that display typical histone modifications of enhancer regions [25,26,27]. Here, we demonstrate that CHD8 is required for the activation of PR enhancers. In proliferating T47D breast carcinoma cells, CHD8 is mostly associated with promoters. However, upon progesterone treatment, CHD8 was quickly recruited to a subset of transcriptionally competent PR enhancers. In agreement with these data, depletion of CHD8 strongly impaired progesterone-regulated gene expression. CHD8 recruitment to the enhancers was dependent of PR but independent of the pioneering factor FOXA1. Interestingly, depletion of the SWI/SNF complex ATPases, BRG1 and BRM, impaired CHD8 recruitment. Furthermore, we observed that CHD8 interacts with the SWI/SNF complex. CHD8 was not required for H3K27 acetylation, but depletion of CHD8 impaired hormone-dependent RNAPII recruitment at enhancers and synthesis of enhancer RNAs (eRNAs), suggesting that CHD8 is required for enhancer transcription.

## Results

### Characterization of genome-wide CHD8 occupancy under normal growth conditions

**Fig 1:**
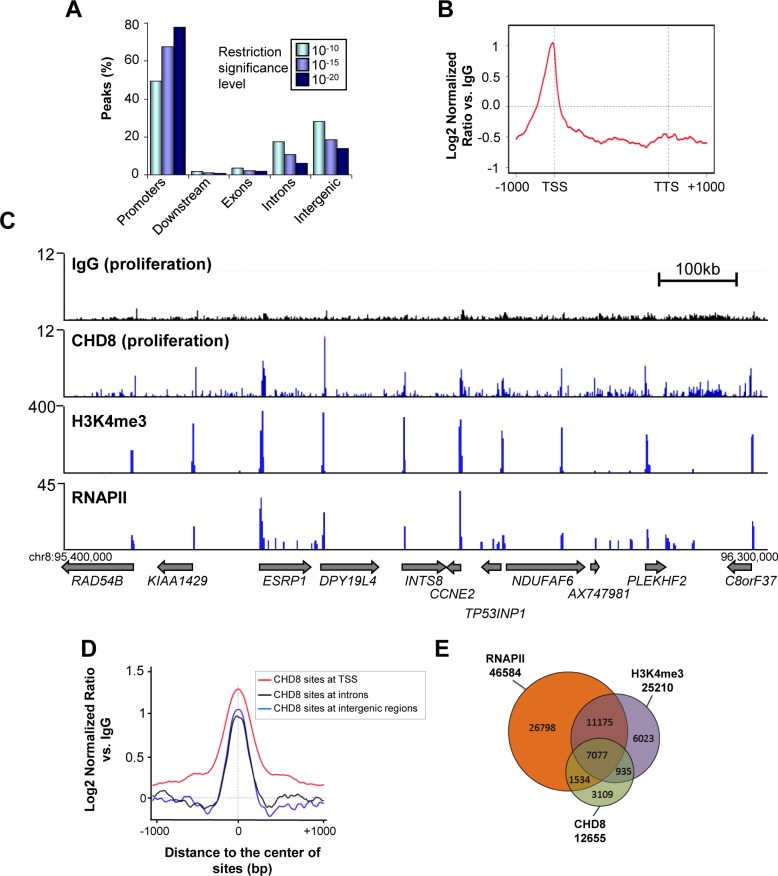
Genome-wide analysis of CHD8 binding sites under normal proliferation conditions. (A) Distribution of CHD8 peaks at low (P < 10^–10^), middle (P < 10^–15^), and high (P < 10^–20^) confidence thresholds, in proliferating T47D-MTVL cells relative to known RefSeq genes. Promoters: ± 2 kb around transcription start site (TSS); Downstream extremities: ± 2 kb around transcription end site; Exons: exonic regions; Introns: intronic regions; Intergenic > 2 kb away from RefSeq TSS. (B) Meta-gene representation of CHD8 ChIP-seq signal at the low confidence threshold. Log2 normalized ratios versus IgG signal are represented. (C) Genome Browser view of IgG, CHD8, H3K4me3 and RNAPII occupancy in a region of chromosome 8. Numbers in the y-axis are reads per million mapped reads. (D) CHD8 occupancy around the centre of the CHD8 binding sites at TSS (red), introns (black) or intergenic regions (blue). (E) Overlapping between CHD8 identified peaks at the low confidence threshold (12655), RNAPII peaks (46586) and H3K4me3 peaks (25210).

To identify the genome-wide distribution of CHD8 in proliferating human breast cancer cells T47D-MTVL [28], we performed chromatin immunoprecipitation of CHD8 followed by deep sequencing (ChIP-seq). We found 12655, 4900 and 2500 peaks of CHD8 by using three different confidence threshold values, respectively (ChIPseeqer threshold level 10^–10^, 10^–15^ and 10^–20^). In all cases, false discovery rates (FDR) were lower than 0.03 (see Materials and Methods). At the lowest threshold about 48% of the peaks (6257) were located within promoters, with a strong enrichment around TSS (Fig 1A and 1B) and about 50% of the peaks were distributed between introns (2187) and intergenic regions (3748). Interestingly, if confidence threshold for peaks identification is increased, the percentage of peaks associated with promoters raise to about 78% (Fig 1A). This is due to differences in CHD8 signal intensity. In fact, CHD8 signals at TSSs are higher than at intergenic or intronic regions (Fig 1C and 1D). Therefore, when only highly significant peaks are analyzed most of them map at promoters. Consistently, a strong overlap between CHD8 and RNA polymerase II (RNAPII) or H3K4me3 ChIP-seq signals was observed (Fig 1C and 1E). CHD8 occupancy was confirmed by ChIP-qPCR in three selected target promoters (*CCND1*, *HDS11B2* and *CCNE2*) using a different anti-CHD8 antibody (S1A Fig). Furthermore, as control, we also verified that knockdown of CHD8 decreased ChIP-qPCR signal (S1B Fig). All these experiments validated our ChIP-seq results. The rest of the analysis was performed using the 12655 CHD8 sites identified at the lowest, but still very significant, threshold. We have previously reported that CHD8 binds 1965 promoters in a ChIP-on-chip analysis of proliferating cervical carcinoma C33 cells [17]. Approximately 60% of the promoters identified as CHD8 targets by ChIP-on-chip (1175) were also identified by ChIP-seq, despite the different cell lines used. CHD8 bound genes were enriched in Gene Ontology categories related to macromolecular biosynthetic processes (transcription, mRNA processing) and cell cycle (S2A Fig). Consistently with our previous data from C33 cells [17] CHD8 target promoters were strongly enriched in E2F (p-value = 3.6 × 10^–135^), ELK-1 (p-value = 2.2 × 10^–122^), AP-2 (p-value = 4.7 × 10^–100^), and SP1 (p-value = 4.2 × 10^–80^) transcription factors binding sites (S2B Fig). Taken together these results indicate that CHD8 is mostly bound to promoters in proliferating T47D-MTVL cells.

### CHD8 is recruited to PR binding sites after progesterone stimulation

**Fig 2:**
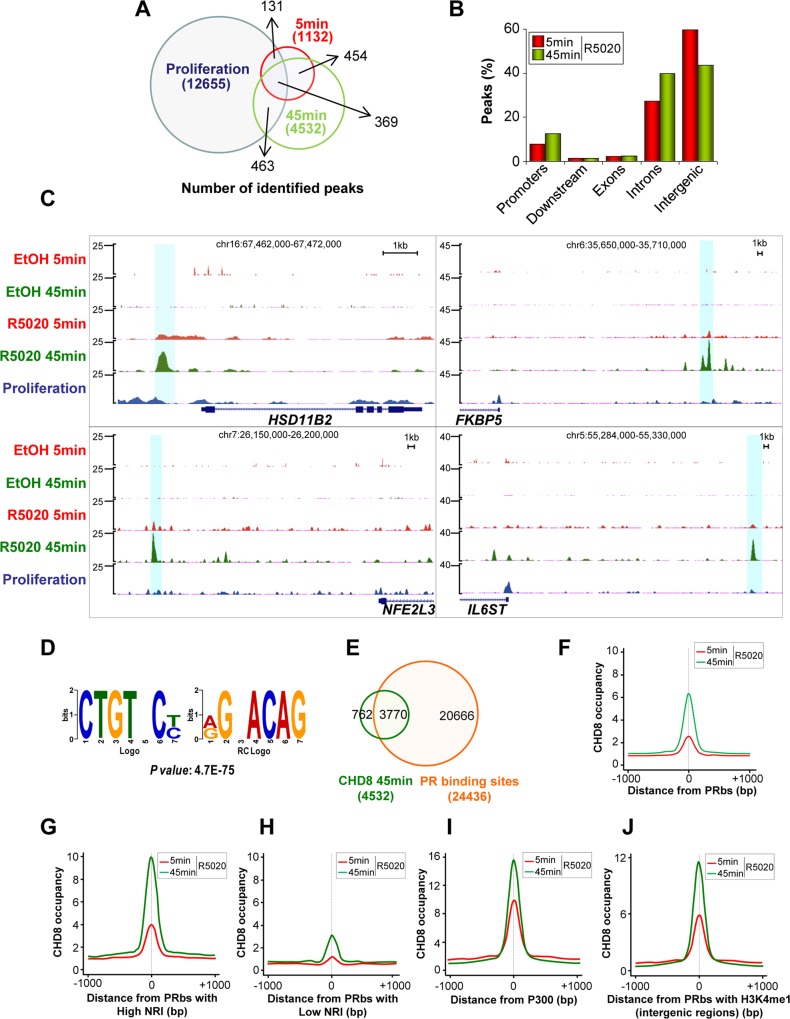
Hormone-dependent CHD8 recruitment to PR binding sites. (A) Overlapping between CHD8 identified peaks in proliferating cells (blue) or in cells stimulated with R5020 for 5 min (red) or 45 min (green). (B) Distribution of CHD8 peaks in cells stimulated with R5020 for 5 or 45 min. Categories as in Fig 1A. (C) Enrichment of CHD8 binding in response to R5020 or vehicle, upon treatment for 5 min (R5020 5 min, EtOH 5 min, red) or 45 min (R5020 45 min, EtOH 5 min, green) or in proliferating un-induced conditions (proliferation, blue), in four regions containing progesterone-responsive genes: *HSD11B2*, *FKBP5*, *NFE2L3* and *IL6ST*. (D) Most significant *de novo* motif (*P*-value: 4.7x10^-75^) identified using ChIPseeqerFIRE and MEME suite [72,73], in the CHD8-binding regions of T47D-MTVL cells stimulated with R5020 for 45 min. (E) Overlapping between progesterone-dependent CHD8 binding sites (green) and PRbs (red) [25] in T47D-MTVL cells stimulated with R5020. (F-J) CHD8 occupancy after 5 (red) or 45 (green) min of R5020 treatment, plotted as the average density of reads counted around the centre of all PRbs (F), around PRbs showing a high (G) or a low (H) nucleosome remodelling index (NRI), around p300 binding sites after R5020 (I) and around PRbs that show H3K4me1 enrichment (J). CHD8 occupancy is expressed as normalized tag density. PRbs, PR binding sites.

CHD8 has been suggested to be a nuclear receptor co-activator [18,19]; however, very little is known about this function of the protein. To gain insight into its role in hormone dependent transcriptional regulation we have analyzed by ChIP-seq the distribution of CHD8 in progesterone-treated T47D-MTVL cells. For that, cells were subjected to 48 h of serum deprivation and then stimulated during 5 or 45 minutes with the synthetic progestin R5020 (10 nM) or the vehicle (ethanol) as control. A very small number of peaks were found in vehicle treated cells, suggesting that serum deprivation strongly decreases the association of CHD8 to the chromatin. These data extend our previous observation about absence of CHD8 in four G1/S transition genes in quiescent cells [17]. However, 1132 and 4532 progestin-induced CHD8 peaks were found at 5 and 45 minutes, respectively, suggesting that CHD8 is quickly recruited to the chromatin after progestin treatment. Most of the sites found after 5 minutes (73%) were also identified after 45 minutes (Fig 2A). Only 18% (832) of the hormone-specific peaks were also found under proliferating conditions (Fig 2A). A large majority of the hormone specific sites were found at intronic and intergenic regions (Fig 2B) and low enrichment was found at TSS (S3 Fig). A representative example of the ChIP-seq data close to four well-known progesterone-dependent genes (*HSD11B2*, *FKBP5*, *NFE2L3* and *IL6ST*) is shown in Fig 2C.

De novo analysis of sequence motifs in progestin-dependent CHD8 bound regions identified a highly significant enrichment (p-value = 4.7 × 10^–75^) for the sequence CTGTNC, which is very similar to the consensus sequence of the progesterone receptor binding site TGTYCY [25] (Fig 2D). PR binds 24436 sites in T47D-MTVL cells 60 min after progestin treatment [25]. Interestingly, 83.2% of the CHD8 progestin-dependent peaks (3770) co-localized with PR peaks, indicating that CHD8 is recruited to a subset of PRbs upon hormone induction (Fig 2E). Thus, CHD8 was significantly enriched around PRbs (Fig 2F). Ballaré et al. have recently reported that PRbs present high nucleosome occupancy and that functional PR sites, involved in transcription control, display a high nucleosome-remodelling index (NRI) [25]. NRI is the ratio between the nucleosome occupancy before and after hormone administration. Strikingly, we found strong CHD8 occupancy at PRbs with high NRI (top 10% higher NRI) and weak CHD8 enrichment at PRbs with low NRI (top 10% lower NRI), suggesting that CHD8 is associated with functional PRbs, where a strong nucleosome remodelling is occurring (Fig 2G and 2H). Similarly to CHD8 binding sites, PRbs are mostly found in introns and intergenic regions [25]. Distal regulatory regions and enhancers are enriched in the histone acetyltransferase p300 [29]. In the presence of R5020, CHD8 signal was strongly enriched around p300 binding sites (Fig 2I), indicating that CHD8 binding sites display enhancer characteristics. Another typical enhancer feature is the presence of monomethylated histone H3 lysine 4 (H3K4me1) [30]. CHD8 was moderately enriched in all regions with high H3K4me1 (S4 Fig). Most interestingly, we observed strong enrichment of CHD8 around H3K4me1 containing PRbs (Fig 2J). Taken together all these data indicate that CHD8 binds functional PR enhancers in a hormone-dependent manner.

### A small percentage of CHD8 sites are co-occupied by CTCF

It has been reported that CHD8 interacts and cooperates with the insulator factor CTCF (CCCTC-binding factor) [11]. Therefore, we have studied the co-localization of both factors under normal growth conditions or after progesterone stimulation. In proliferating T47D-MTVL cells about 16.5% (p-value = 6.0 x 10^–130^, hypergeometric distribution) of the CHD8 containing regions were also enriched in CTCF (S5A Fig). However, this was only 4.4% of the CTCF sites. CTCF mostly binds to intergenic or intronic regions [31]. Consistently, most of the CTCF-CHD8 co-occupied sites (66%) were also at intergenic and intronic regions (S5B Fig). Upon progesterone stimulation only about 5.4% of the CHD8 sites and 0.6% of the CTCF sites are co-occupied by both factors (S5C Fig), suggesting that CHD8 and CTCF do not cooperate for progesterone-mediated regulation.

### CHD8 is involved in progesterone-dependent gene regulation

**Fig 3:**
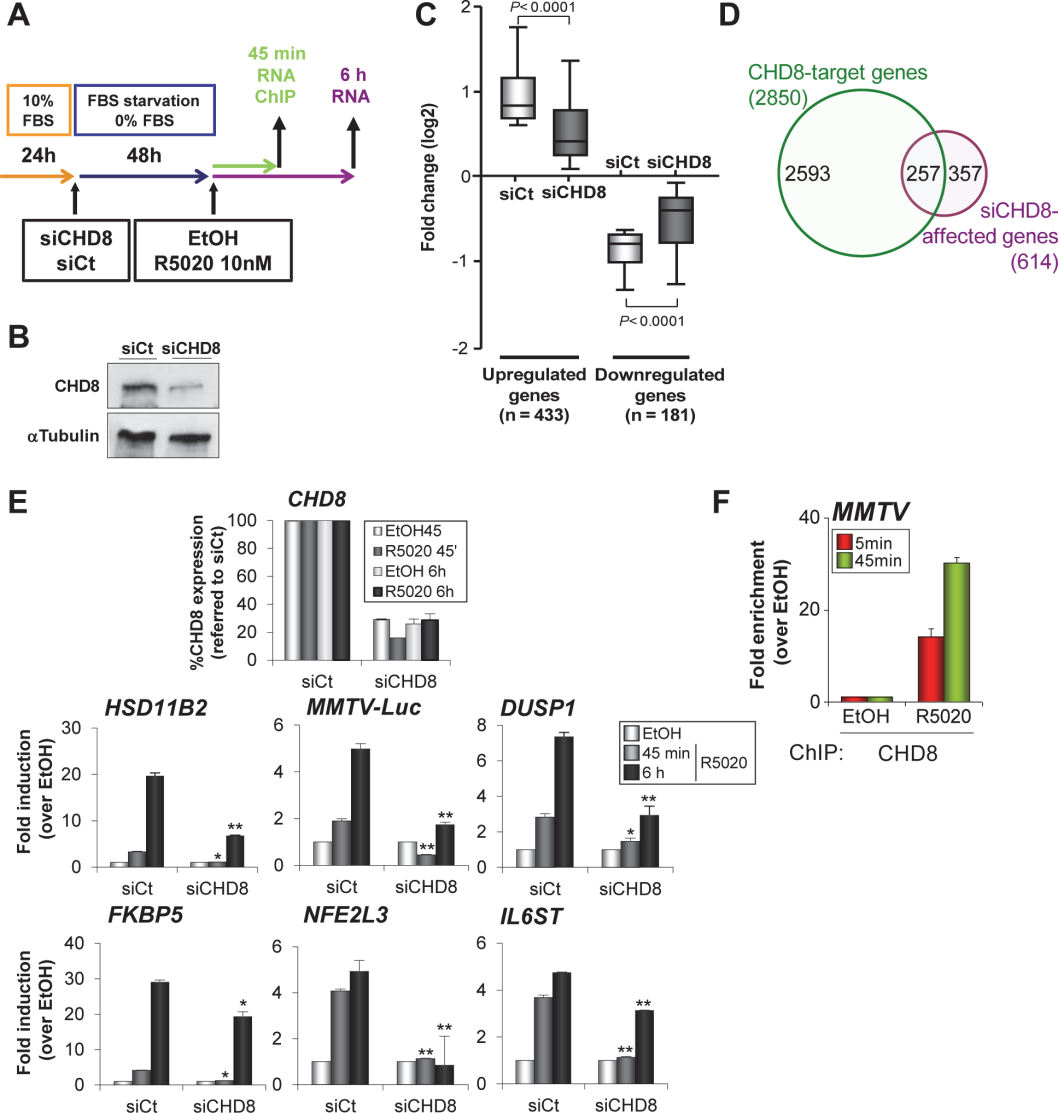
CHD8 is required for progesterone-dependent gene regulation. (A) Flow diagram depicting the knockdown strategy for CHD8 and the hormone treatment in gene expression and ChIP studies. T47D-MTVL cells were transfected with control siRNA (siCt) or siRNA against CHD8 (siCHD8), subjected to serum deprivation during 48 h and then stimulated with 10 nM R5020 (R5020) or vehicle (EtOH) for 45 min or 6 h, depending on the experiment. (B) Western blot analysis of CHD8 expression upon transfection of T47D-MTVL cells with control siRNA (siCt) or siRNA against CHD8 (siCHD8). (C) Box-and-whisker plots of the change in gene expression of CHD8-dependent genes (see Materials and Methods) after 6 h of R5020 treatment. siCHD8: cells depleted of CHD8; siCt, control cells. (D) Overlapping between R5020-dependent CHD8-target genes (green) and genes that are differentially regulated in response to R5020 in CHD8-silenced cells with respect to control cells (see Materials and Methods) (purple, siCHD8-affected genes). Chip-seq CHD8 peaks were assigned to the closer gene. (E) Effect of CHD8 depletion in progestin-dependent expression of the following genes: *HSD11B2*, *MMTV-Luc*, *DUSP1*, *FKBP5*, *NFE2L3* and *IL6ST*. Level of *CHD8* expression was determined as control of silencing (upper panel). mRNA levels were determined by RT-qPCR after 45 min or 6 h of stimulation. Data are the mean of at least n = 6 qPCR reactions from three independent experiments. Error bars represent ± SD values. * *p* < 0.001; ** *p* < 0.0001 with respect to siCt, using Student’s t-test. (F) ChIP analysis of CHD8 occupancy at the MMTV regulatory region upon stimulation with R5020 during 5 or 45 min. Data are the mean of at least n = 6 qPCR reactions from three independent experiments.

To correlate CHD8 binding sites with CHD8-regulated gene expression we performed a transcriptomic analysis of T47D-MTVL cells transfected with a control siRNA or a siRNA specifically targeting CHD8 and stimulated during 6 h with progestin or vehicle (Fig 3A and 3B). We found 1170 genes differentially expressed (FDR< 0.01 and lineal change > 1.5 fold) after progestin treatment of control cells, of which 793 were up-regulated and 377 down-regulated. About 52.5% of these genes (614) were misregulated in CHD8-depleted cells with respect to control cells. Interestingly, CHD8-dependent genes presented lower induction of up-regulated genes and lower repression of down-regulated genes, indicating that CHD8 is required for progesterone-dependent regulation of a subset of genes (Fig 3C). Consistently, around 42% of the CHD8-dependent genes (257) were found to be close to CHD8 genomic locations (P = 9.96 x 10^–103^) (Fig 3D).

Next, we verified by RT-qPCR that CHD8 was required for normal progestin-dependent induction of several well known progesterone dependent genes that contain close hormone-dependent CHD8 binding sites, such as *HSD11B2*, *DUSP1*, *FKBP5*, *NFE2L3* and *IL6ST* genes. Thus, depletion of CHD8 severely impaired accumulation of mRNA, both 45 min and 6 h after progestin treatment (Fig 3E). As a control, we confirmed that a different siRNA that targets CHD8 had similar effects on expression of progesterone dependent genes (S6 Fig). T47D-MTVL cells contain a single copy of the MMTV-*Luc* transgene integrated in their genome [28]. CHD8 was also strongly enriched in a progestin-dependent manner at the MMTV-*Luc* transgene promoter (Fig 3F). Furthermore, depletion of CHD8 severely impaired induction of MMTV-*Luc* (Fig 3E and S6 Fig). In summary, these data demonstrate that CHD8 is necessary for progestin-dependent regulation, at least in a subset of target genes.

### PR is required for CHD8 recruitment to chromatin upon progestin stimulation

**Fig 4:**
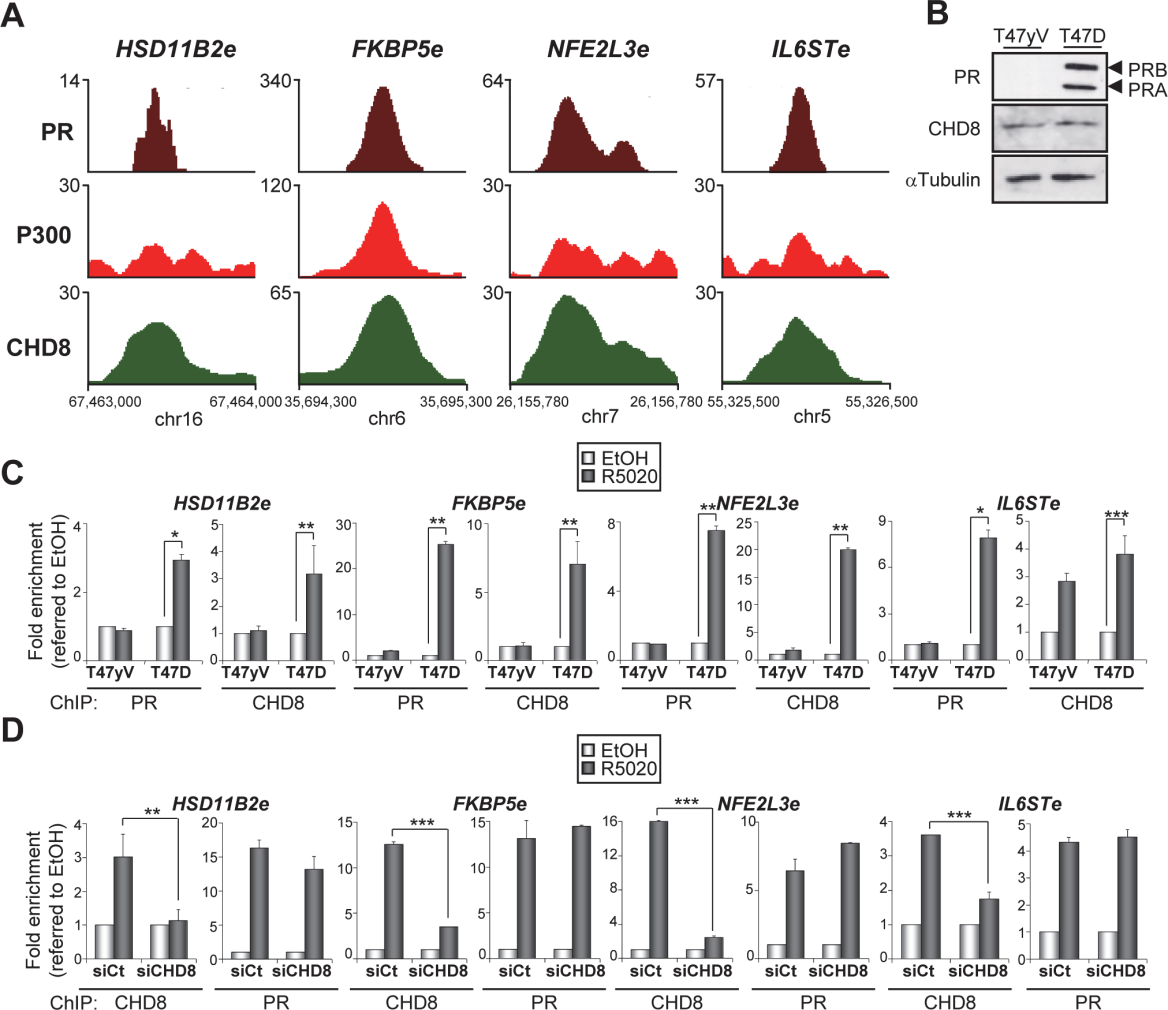
PR is necessary for hormone-dependent CHD8 recruitment to PR enhancers. (A) ChIP-seq binding profile for PR, P300 and CHD8 upon R5020 treatment at the indicated enhancer regions. Numbers in the y axis indicates number of reads, while numbers in the x axis indicate chromosomal position. (B) PR and CHD8 expression in T47-YV or T47D-MTVL was analyzed by Western blotting with anti-PR and anti-CHD8 antibodies. (C) ChIP analysis of PR and CHD8 occupancy at the indicated enhancers in T47-YV or T47D-MTVL cells stimulated with R5020 (R5020) or vehicle (EtOH) for 45 min. (D) ChIP analysis of CHD8 and PR at the indicated enhancers in T47D-MTVL cells transfected with control siRNA (siCt) or siRNA against CHD8 (siCHD8), and then stimulated with R5020 (R5020) or vehicle (EtOH) for 45 min. (C, D) Data are the mean of at least n = 6 qPCR reactions from three independent experiments. Error bars represent ± SD values. * *p* < 0.02; ** *p* < 0.01; *** *p* < 0.001 using Student’s t-test.

Next we selected four CHD8 peaks close to *HSD11B2*, *FKBP5*, *NFE2L3* and *IL6ST* genes that display high ChIP-seq enrichment for CHD8 and PR upon progestin stimulation (Fig 4A). These regions were also enriched for the typical enhancer factor p300 (Fig 4A). Therefore, we called these regions *HSD11B2e*, *FKBP5e*, *NFE2L3e* and *IL6STe*. All enhancers were located upstream of the corresponding genes (Fig 2C). Then, we decided to investigate how PR affects CHD8 recruitment to these enhancers and vice versa. First we analyzed PR and CHD8 recruitment to CHD8 progestin-dependent peaks in T47D-MTVL cells or in T47D-YV, a PR-negative clonal derivative cell line of T47D [32] (Fig 4B). We verified by western blotting that levels of CHD8 were identical in T47D-MTVL and in T47D-YV (Fig 4B). ChIP experiments performed 45 minutes after progestin stimulation confirmed that high levels of PR are recruited to all analyzed regions in T47D cells but, as expected, not in T47D-YV cells (Fig 4C). Interestingly, CHD8 was also strongly recruited to all analyzed regions in T47D-MTVL cells but not in T47D-YV cells, suggesting that PR is required for hormone-dependent CHD8 recruitment to PRbs (Fig 4C). A significant recruitment of CHD8 to the *IL6ST* regulatory region was observed in T47D-YV cells suggesting that CHD8 is recruited to this region, at least in part, independently of PR. Residual binding of progestin to other nuclear receptor might be responsible of this effect.

Next, we determined whether CHD8 is involved in PR recruitment to PRbs in T47D-MTVL cells. Fig 4D shows that CHD8 depletion (siCHD8) did not affect progestin-dependent PR recruitment to the four analyzed regulatory regions. As a control, we verified that silencing of CHD8 strongly decreased its association with chromatin, validating the ChIP signals. Taken together, these data indicate that PR is required for CHD8 recruitment to progesterone-dependent CHD8 binding sites, but PR does not require CHD8 for binding.

### Depletion of FOXA1 stimulates PR and CHD8 recruitment to PRbs

**Fig 5:**
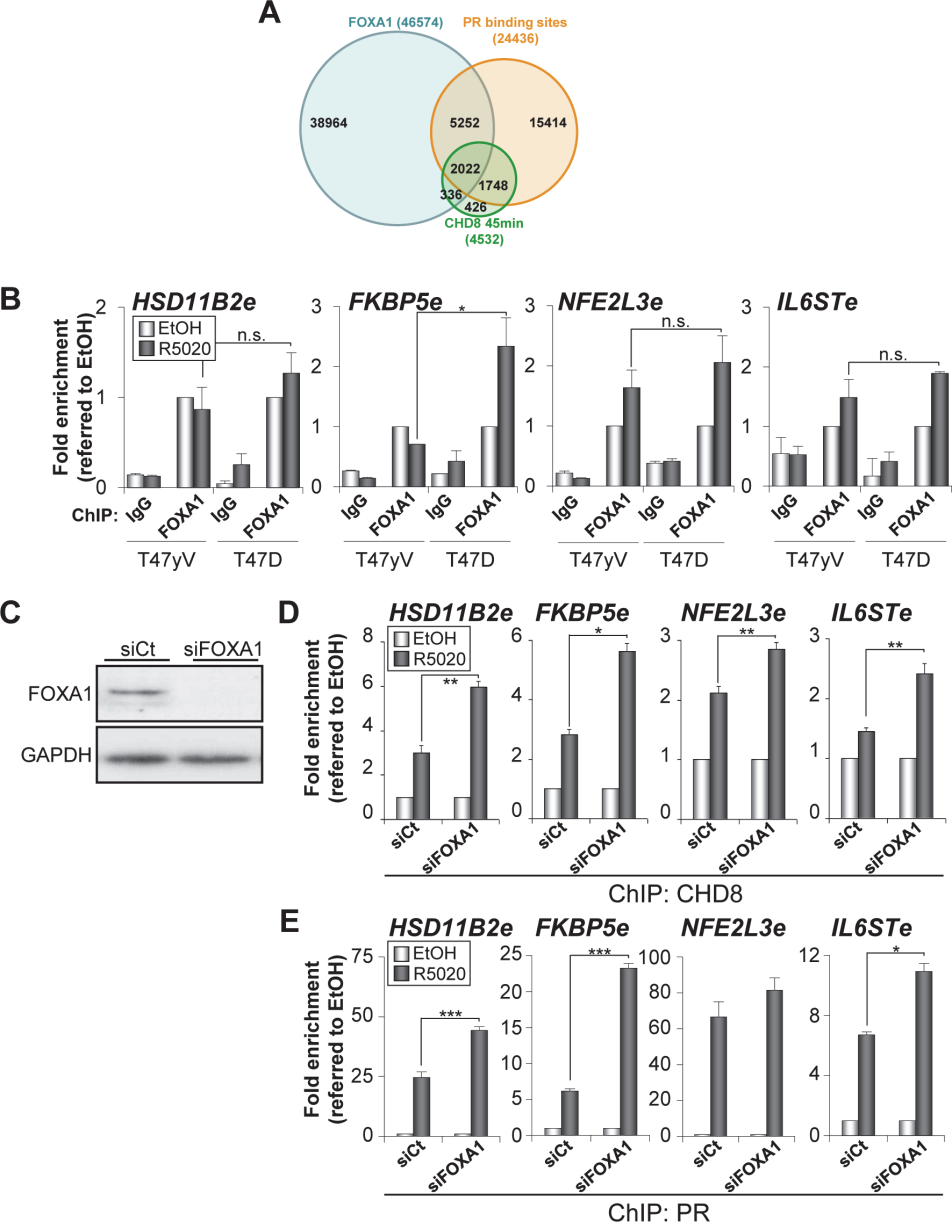
Depletion of FOXA1 stimulates PR and CHD8 recruitment to PRbs. (A) Overlapping between progesterone-dependent CHD8 binding sites (green), progesterone-dependent PRbs (red) [25] and FOXA1 binding sites (blue) in T47D cells (ENCODE dataset GSM803409). (B) ChIP analysis of FOXA1 occupancy at the indicated enhancers in T47-YV or T47D-MTVL cells stimulated with R5020 (R5020) or vehicle (EtOH) for 45 min. (C) Western blot analysis of FOXA1 expression upon transfection of T47D-MTVL cells with control siRNA (siCt) or siRNA against FOXA1 (siFOXA1). (D-E) ChIP analysis of CHD8 (D) and PR (E) at the indicated enhancer in T47D-MTVL cells transfected with control siRNA (siCt) or siRNA against FOXA1 (siFOXA1), and then stimulated with R5020 (R5020) or vehicle (EtOH) for 45 min. (B, D-E) Data are the mean of at least n = 6 qPCR reactions from three independent experiments. Error bars represent ± SD values. * *p* < 0.01; ** *p* < 0.001; *** *p* < 0.0001 using Student’s t-test; n.s., not significant.

FOXA1 is a fork-head family transcription factor able to directly bind to DNA in the surface of a nucleosome [33]. Because of this pioneering ability, FOXA1 is able to facilitate estrogen receptor binding to the chromatin of hormone-dependent enhancers [34,35]. Since PR is able to bind directly to nucleosomes [28], it is unclear whether FOXA1 also cooperates with PR. ChIP-seq data of FOXA1 distribution in unstimulated T47D cells are available from ENCODE. Using these data we have observed that 52% (2358) of the CHD8 binding sites were also enriched for FOXA1 (Fig 5A). Furthermore, 54% (2022) of the CHD8-PR co-occupied regions were also occupied by FOXA1. Given this very significant enrichment (P = 1.17 x 10^–173^) we decided to study the role of FOXA1 in CHD8 and PR recruiting. First, we investigated using ChIP whether FOXA1 binds to the analyzed regulatory regions of *HSD11B2*, *FKBP5*, *NFE2L3* and *IL6ST* genes. As shown in Fig 5B significant levels of FOXA1 were found at all studied regions. FOXA1 occupancy after progestin treatment at *HSD11B2e*, *NFE2L3e* and *IL6STe* enhancers was similar in T47D-MTVL and T47D-YV cells, suggesting that PR is not involved in FOXA1 recruitment at these regions (Fig 5B). However, a possible role of ligand-bound PR in FOXA1 recruitment was observed at the *FKBP5e* enhancer (Fig 5B).

Next, we determined the effect of FOXA1 silencing (Fig 5C) on the hormone-dependent recruitment of CHD8 and PR in T47D-MTVL cells. Surprisingly, depletion of FOXA1 significantly increased the level of occupancy of both PR and CHD8 at the four regulatory regions analysed (Fig 5D and 5E), suggesting that, at least in these progestin-dependent regulatory regions, FOXA1 does not help PR and subsequent CHD8 recruitment. Moreover, our data suggest that FOXA1 might compete with PR for binding to PR-enhancers.

### CHD8 interacts with human SWI/SNF complexes

**Fig 6:**
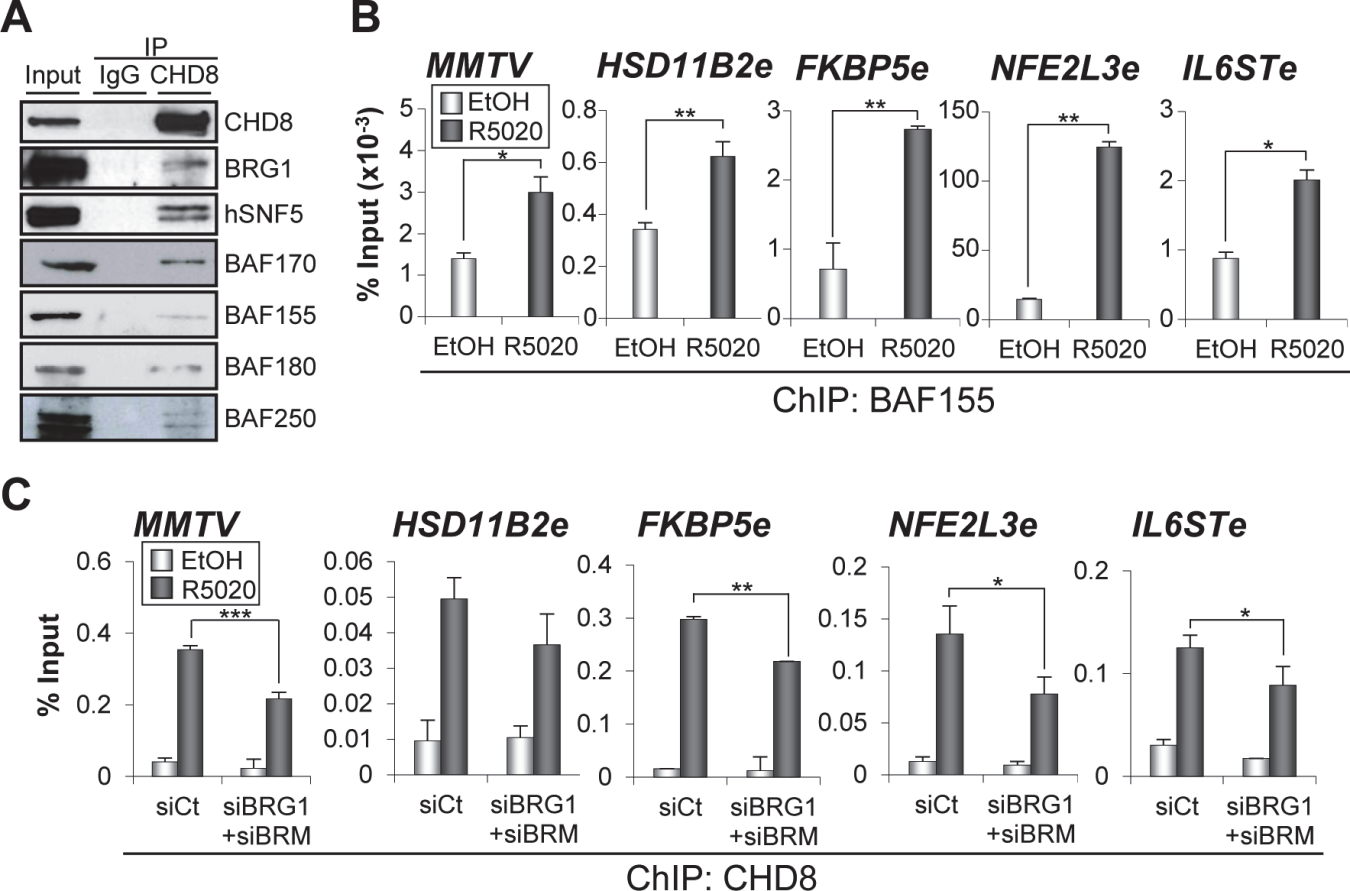
SWI/SNF complexes interact with CHD8 and are involved in CHD8 recruitment. (A) SWI/SNF subunits co-immunoprecipitate with CHD8. Extract from T47D-MTVL cells were subjected to immunoprecipitation using anti-CHD8 antibody. Precipitated proteins were then revealed by western blotting using the indicated antibodies against BAF or PBAF subunits. (B) ChIP analysis of BAF155 at the indicated regions in T47D-MTVL cells stimulated with R5020 (R5020) or vehicle (EtOH) for 45 min. (C) ChIP analysis of CHD8 at the indicated regions in T47D-MTVL cells transfected with control siRNA (siCt) or siRNA against BRM and BRG1 (siBRM+siBRG1), and then stimulated with R5020 (R5020) or vehicle (EtOH) for 45 min. (B, C) Data are the mean of at least n = 6 qPCR reactions from three independent experiments. Error bars represent ± SD values. * *p* < 0.05; ** *p* < 0.01; *** *p* < 0.001 using Student’s t-test.

PBAF and BAF are closely related chromatin remodelling complexes of the SWI/SNF family, which share multiple protein subunits. The BAF complex is required for progesterone-dependent gene activation [21,22,25]. Furthermore, BAF250 and BAF57, two of the subunits of the complex, interact with PR in a hormone-dependent manner [22]. Then, we decided to investigate whether CHD8 interacts with the SWI/SNF complexes. For that, we performed immunoprecipitation using anti-CHD8 antibodies from extracts of T47D-MTVL cells. CHD8 co-precipitated with the core subunits INI1/hSNF5/BAF47, BAF170 and BAF155 and with the ATPase BRG1 in T47D-MTVL cells (Fig 6A). All these subunits form part of both SWI/SNF complexes: BAF and PBAF. To identify the type of SWI/SNF complex interacting with CHD8, we also investigated the presence of BAF180, a PBAF-specific subunit, and BAF250, a BAF-specific subunit. As shown in Fig 6A both subunits co-precipitated with CHD8 indicating that CHD8 can interact with both complexes. The CHD8-SWI/SNF interaction was found both, in the presence and in the absence of hormone (S7A Fig). Next, we studied whether the SWI/SNF complex is required for CHD8 recruitment to PRbs. First, we verified by ChIP that BAF155, one of the core subunits of the complex, is recruited to the four analyzed CHD8-bound enhancers and to the MMTV promoter (Fig 6B). Then, we demonstrated that knockdown of BRG1 and BRM (S7B and S7C Fig) significantly impaired recruitment of CHD8 to the four analyzed PR enhancers and to the MMTV promoter (Fig 6C). Taken together, these data suggest that CHD8 interacts with the SWI/SNF complex and that this interaction contributes to recruit or to stabilize CHD8 at PRbs.

### CHD8 is not required for H3K27 acetylation but contributes to reorganize the chromatin of enhancer regions

**Fig 7:**
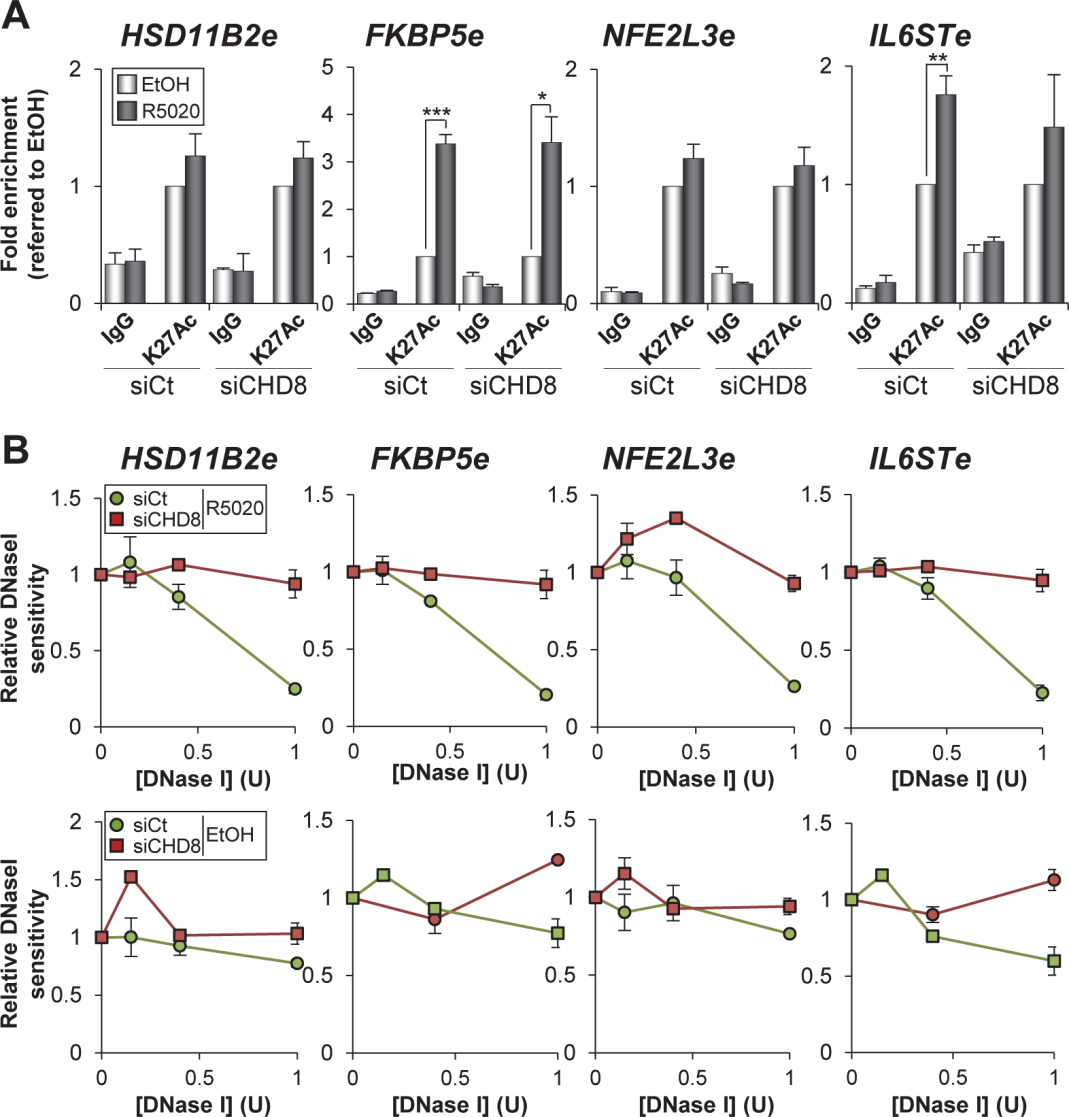
CHD8 involvement in modification of enhancer chromatin. (A) CHD8 is not required for H3K27 acetylation. ChIP analysis of H3K27Ac enrichment at the indicated enhancers in T47D-MTVL cells transfected with control siRNA (siCt) or siRNA against CHD8 (siCHD8), and then stimulated with R5020 (R5020) or vehicle (EtOH) for 45 min. Data are the mean of at least n = 6 qPCR reactions from three independent experiments. Error bars represent ± SD values. * *p* < 0.01; ** *p* < 0.001; *** *p* < 0.0001 using Student’s t-test. (B) CHD8 contributes to open chromatin at PR enhancers. DNase I sensitivity at the indicated regions in T47D-MTVL cells transfected with control siRNA or siRNA against CHD8, and then stimulated with R5020 or vehicle for 45 minutes. Error bars represent ± SD (n = 3).

Presence of H3K27Ac distinguishes active enhancer states from those poised for activation [36,37,38]. H3K27 is acetylated by p300 [39] and we have shown that CHD8 is enriched around p300 binding sites (Fig 2I). To further characterize the role of CHD8 in enhancer activation we investigated whether CHD8 affects the level of H3K27Ac at the four selected CHD8 binding sites. At the *FKBP5e* and *IL6STe* regions H3K27 acetylation was enhanced by R5020 (Fig 7A). However, significant levels of H3K27Ac were already observed under un-stimulated conditions at the *HSD11B2e* and *NFE2L3e* enhancers, which were not further stimulated by progestin (Fig 7A). Interestingly, CHD8 depletion did not affect the level of H3K27Ac at any of the analyzed regions, suggesting that CHD8 is not involved in p300 recruiting or H3K27 acetylation.

Next we investigated whether CHD8 is required to open the chromatin of its target enhancers. For that, we performed quantitative DNase I sensitivity assays in *HSD11B2e*, *FKBP5e*, *NFE2L3e* and *IL6STe* enhancer regions in the presence of hormone or vehicle, as described in [40]. Interestingly, CHD8 knockdown decreased hormone-dependent accessibility to the enzyme of all the analyzed regions suggesting that CHD8 is involved in the hormone-dependent chromatin remodelling of these regions (Fig 7B).

### CHD8 is required for RNAPII recruitment and eRNA synthesis at PR enhancers

**Fig 8:**
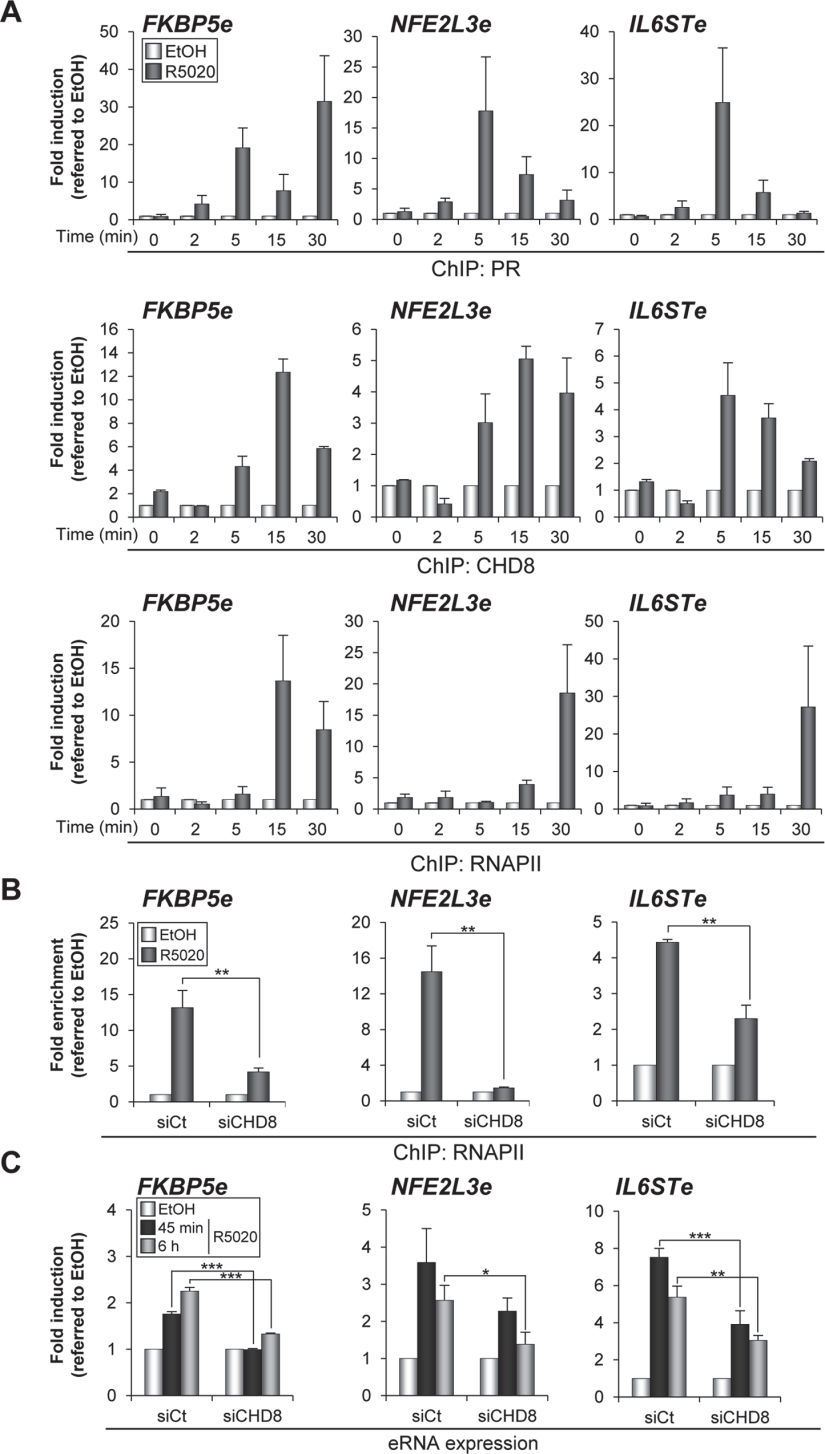
CHD8 is required for RNAPII recruitment and eRNA synthesis at PR enhancers. (A) Time course analysis of PR, CHD8 and RNAPII recruitment to *FKBP5e*, *NFE2L3e* and *IL6STe* enhancers by ChIP. T47D-MTVL cells were stimulated with R5020 (R5020) or vehicle (EtOH) for the indicated times and then processed for ChIP by using antibodies against PR, CHD8 and RNAPII. (B) ChIP analysis of RNAPII enrichment at the indicated enhancers in T47D-MTVL cells transfected with control siRNA (siCt) or siRNA against CHD8 (siCHD8), and then stimulated with R5020 (R5020) or vehicle (EtOH) for 45 min. (C) Expression of enhancer RNAs (eRNA) from the indicated enhancers in T47D-MTVL cells transfected with control siRNA (siCt) or siRNA against CHD8 (siCHD8), and then stimulated with R5020 (R5020) or vehicle (EtOH) for 45 min or 6 h. (A-C) Data are expressed as fold induction relative to the level in ethanol treated cells. Data are the mean of at least n = 6 qPCR reactions from three independent experiments. Error bars represent ± SD values. * *p* < 0.01; ** *p* < 0.001; *** *p* < 0.0001 using Student’s t-test.

Several studies have shown that many active enhancers present significant occupancy of RNAPII and regulated production of bidirectional RNA, called eRNAs [41,42,43,44,45]. We have investigated whether RNAPII is recruited to *FKBP5e*, *NFE2L3e* and *IL6STe* enhancer regions and the order of recruitment with respect to PR and CHD8. For that, binding of PR, CHD8 and RNAPII at 0, 2, 5, 15 and 30 min after progestin stimulation was determined by ChIP-qPCR (Fig 8A). As previously reported for the MMTV promoter [21], PR was found at the enhancers as early as 2 min after hormone addition. CHD8 was absent at 2 min but was found at the 5 min time point, remaining in the enhancers during the 15 and 30 min time points. Finally, RNAPII occupancy increased between 15 and 30 min at the *FKPB5e* and only at 30 min at *NFE2L3e* and *IL6STe* enhancers (Fig 8A). These data indicate that RNAPII is recruited to the enhancers after PR and CHD8 and suggest that RNAPII recruitment is a late event during the process of enhancer activation. Then, we investigated the role of CHD8 in RNAPII recruitment. Interestingly, hormone-stimulated RNAPII occupancy was strongly impaired in CHD8-depleted cells (Fig 8B).

Next, eRNA synthesis at *FKBP5e*, *NFE2L3e* and *IL6STe* enhancers was evaluated by RT-qPCR both 45 min and 6 h upon R5020 addition (see Materials and Methods). Progestin increased between 2 and 7 fold production of eRNAs already at 45 min and expression was maintained after 6 h (Fig 8C). As a control, we verified that no qPCR signal was observed in the absence of reverse transcriptase. Consistently with the effect of CHD8 depletion in RNAPII occupancy, silencing of CHD8 significantly impaired eRNA synthesis from the three analyzed regions (Fig 8C). These results indicate that CHD8 is required for RNAPII recruitment and enhancer transcription, at least from a subset of PR enhancers.

## Discussion

Steroid hormone transcriptional regulation requires binding of nuclear receptors to thousand of binding sites that mostly map in intergenic and intronic regions and that show features of transcription enhancers. The recent discovery of enhancer-associated transcripts opens the door to investigate how enhancer transcription is controlled. We, and others, have previously found that the chromatin remodeler CHD8 is found at promoters [9,13,14,16,17,19,46]. The data presented in this manuscript demonstrate that shortly after progestin stimulation of quiescent cells, CHD8 binds progesterone enhancers. Consistently, depletion of CHD8 impairs progestin-dependent transcriptional response. CHD8 recruiting requires PR, but not the pioneering factor FOXA1. We also show that CHD8 interacts with the SWI/SNF complex and that SWI/SNF is important for normal CHD8 recruitment. Furthermore, we demonstrate that CHD8 is not required for acetylation of H3K27 in enhancers, but it is necessary for DNase I accessibility, for normal recruitment of RNAPII and for the synthesis of progestin-dependent eRNAs, suggesting that CHD8 plays a role in late phases of progesterone enhancers activation.

### CHD8 binds promoters and enhancers

We have recently reported a promoter ChIP-on-chip analysis demonstrating that, in cervix carcinoma C33 cells, CHD8 binds around 2000 active promoters also enriched in H3K4me2 and H3K4me3, and that it is required for expression of E2F-dependent G1/S transition genes [17]. In agreement with these results, our genome-wide ChIP-seq analysis reveals that most of CHD8 is bound to promoters also enriched in RNAPII and H3K4me3 in proliferating T47D-MTVL cells. While this paper was in revision another group has also shown that CHD8 binds thousand of active promoters in an iPSC-derived neuronal progenitor cell line [47]. All these data from different cell lines support that CHD8 is a promoter-associated factor. However, upon progestin stimulation CHD8 was rarely found at promoters; instead it was recruited to a number of PRbs mostly located in intergenic and intronic regions enriched in H3K4me1 and p300, indicating that these sites are progesterone-dependent enhancers. Interestingly, under normal proliferation conditions, CHD8 was found at the promoters of genes close to these enhancers (p < 0.001). While this fact might suggest that CHD8 dynamically move from enhancers to close promoters, further experiments are required to demonstrate this association. Our data demonstrate that CHD8 binds both, promoters and enhancers upon specific stimulation such as progesterone. CHD8 has also been involved in AR-dependent [19] and ER-dependent [18] regulation. It is, therefore, possible that CHD8 also binds AR or ER enhancers in the presence of the appropriated hormonal stimulus.

CHD7 is a paralogue of CHD8 and both proteins has been shown to interact [48]. ChIP-seq analysis of mice ES cells has demonstrated that CHD7 is mostly associated with a subset of active enhancers and promoters [49,50]. Comparison of CHD8 genomic distribution with that of CHD7 available from ENCODE showed that only 7.1% and 5.6% of the CHD8 peaks overlap with CHD7 containing regions in K562 or H1 stem cells, respectively. Most enhancers are tissue- and cell line-specific and therefore it is difficult to compare genomic positions between different cell lines. Nevertheless, these data suggest that although CHD7 and CHD8 may cooperate in some genomic locations, they also have specific independent roles.

It has been reported that CTCF interacts with the carboxy-terminus of CHD8 and that CHD8 co-occupy several CTCF binding sites [11]. Our genome-wide study demonstrates that, in the absence of progesterone, 16.5% of the CHD8 binding sites are enriched for CTCF and 4.4% of CTCF sites are occupied by CHD8. While this percentage is largely higher than expected by chance, it is clear that CHD8 might only be involved in CTCF function in a small percentage of insulators. Since CTCF sites are often located at enhancer regions [51] and enhancer looping activity has been related to CTCF and cohesins [52], it is possible that the CHD8-CTCF co-occupancy is more related to the role of CHD8 in enhancers than to specific functions in insulation. In the presence of progestin the number of sites co-occupied by CTCF and CHD8 is even lower, suggesting that their association is not related to progesterone dependent transcription.

### How is CHD8 recruited to progesterone enhancers?

Here we show that CHD8 is not recruited to four selected progesterone enhancers in the absence of PR, indicating that PR is essential for the recruiting. This is consistent with our time-course experiment where PR was found at the enhancers earlier than CHD8 upon hormone addition. However, we have been unable to detect direct PR-CHD8 interaction suggesting that CHD8 may be recruited through interaction with other co-regulators of PR enhancers. Despite the very significant overlapping between the pioneering factor FOXA1 and CHD8 binding sites, we show that FOXA1 silencing even increased CHD8 occupancy at the four analyzed enhancers, indicating that FOXA1 is not required for CHD8 recruitment. The chromatin remodelling BAF complex is recruited to PRbs [25] and is required for progesterone-dependent remodelling of the MMTV promoter [21,22]. Interestingly, we have observed that CHD8 interacts with both SWI/SNF complexes: BAF and PBAF. In addition, two high throughput proteomic analysis have identified co-immunoprecipitation of CHD8 and SWI/SNF subunits [53,54]. Since PR directly interacts with BAF complex it is possible that CHD8 is recruited to PRbs through interaction with the BAF complex. Consistently with this hypothesis, knocking down of BRG1 and BRM reduced CHD8 recruiting. Interestingly, it has been reported that the CHD8 paralogue, CHD7, interacts with PBAF, the other human SWI/SNF complex [55]. Whether other CHD8 paralogues, CHD6 and CHD9, also interact with SWI/SNF complexes is unknown. It is also unclear the extent to which CHD8 and SWI/SNF complexes cooperate and whether they can have common and independent targets. It is worth noting that both, SWI/SNF complexes and CHD8 are required for activation of E2F-dependent genes at the G1/S transition [17] [56].

On the other hand, we have reported that the chromodomains of CHD8 binds dimethylated and trimethylated H3K4 peptides [16]. H3K4me2 modification is typically associated with enhancers [57,58,59]. In addition, Vicent et al. reported that H3K4 methylation by the ASCOM (ASC-2 [activating signal cointegrator-2] complex) is required for the progesterone-dependent induction of the MMTV promoter [21]. Therefore, it is possible that CHD8 chromodomains interaction with methylated H3K4 also contributes to the recruitment or the stabilization of CHD8 at PR enhancers.

### How does CHD8 regulate PR enhancers?

We have shown that CHD8 depletion does not impair PR binding. Since PR directly interacts with BAF complex it is unlikely that CHD8 is required for BAF recruitment or activity. In fact, we show that BRG1 and BRM are required for normal CHD8 occupancy of PR enhancers suggesting that CHD8 acts downstream of the BAF complex. Another common step of enhancer activation is acetylation of H3K27 by the histone acetyltransferase p300 [29,36,37,38]. Ballare et al., found that progestin-dependent PR biding sites were enriched in regions that contained p300 under un-stimulated conditions and that, in general, the level of p300 increased upon hormone treatment. We have observed that progestin stimulated H3K27 acetylation at the *FKBP5e* and *IL6STe* enhancers but not at the *HSD11B2e* and *NFE2L3e* enhancers. Depletion of CHD8 did not affect levels of H3K27Ac at any of the regions, suggesting that CHD8 was not involved in this step of enhancer activation.

Numerous reports have evidenced that enhancer activation involves RNAPII recruiting and synthesis of eRNA, monodirectional or bidirectional transcripts of 0.5 to 5 kb. Recent results demonstrate that eRNAs are involved in transcriptional activation of neighbouring genes, in enhancer-promoter looping and in directing chromatin-remodelling events at specific promoters [60,61,62,63,64]. The time-course experiment shown in Fig 8A indicates that RNAPII is recruited between 15 and 30 minutes after hormone stimulation, while PR and CHD8 reach the enhancers around 2 and 5 min after stimulation, respectively. We show that depletion of CHD8 strongly impairs recruiting of RNAPII and synthesis of eRNAs, demonstrating that CHD8 is required for these late events of progesterone enhancers activation. In *Drosophila*, the CHD8 orthologous Kismet is found at transcriptionally active genes in polytene chromosomes [65]. Kismet mutations reduce the level of phosphorylated elongating RNAPII but not the level of initiating RNAPII, suggesting that Kismet is involved in transcription elongation. Human CHD8 interacts with elongating RNAPII [16]. Furthermore, CHD8-depleted cells are hypersensitive to drugs that inhibit phosphorylation of serine 2 of the carboxy-terminal domain (CTD) of POLR2A, the largest subunit of RNAPII (DRB and flavopiridol), an early step of the transcription cycle [16]. These data suggest that, as Kismet, CHD8 may also be involved in elongation. Kaikkonen et al., have recently reported that eRNA synthesis is sensitive to flavopiridol [58], suggesting that eRNA synthesis also requires CTD serine 2 phosphorylation. Therefore, it is possible that CHD8 is required for transcription elongation of eRNAs at PR enhancers.

We also show that CHD8 is required to increase DNase I sensitivity at enhancers upon hormone treatment, suggesting that CHD8 may be involved in the hormone-dependent nucleosomal remodeling that occurs at a subset of PRbs [25]. It is well known that transcription increases DNase I sensitivity of gene bodies [66,67]. Therefore, it is also possible that CHD8 effect on DNase I sensitivity is caused by its role in enhancer transcription.

Vicent et al. demonstrated that BAF and NURF chromatin remodelling machines are required for PR-dependent activation of the MMTV promoter and other PR enhancers [22,25]. Now we add a third actor, CHD8, to the list of remodelers required for progesterone-dependent activation. However, CHD8 seems to be recruited only to at subset of PRbs, including the MMTV promoter. Future experiments will be required to find what determines CHD8 recruitment to some PRbs and not to others. It is worth noting that it has been reported that CHD9 interacts in vitro with nuclear receptors such as PPARA (PPARα), NR1I3 (CAR), NR3C1, ESR1 (ERα) and RXRA [68,69]. So, it is tempting to speculate that other CHD8 paralogues may also regulate, redundantly or non-redundantly with CHD8, hormone-dependent enhancers activation.

## Materials and Methods

### Cell culture and experimental conditions

T47D-MTVL human breast cancer cells carrying one stably integrated copy of the luciferase reporter gene under the control of the MMTV promoter [28] and T47D-YV cells (PR-negative clonal derivative cell line of T47D [32,70]) were routinely grown in RPMI 1640 medium supplemented with 10% FBS, 2 mM L-glutamine, 100 U/ml penicillin and 100 mg/ml streptomycin. Cells were grown exponentially (in 10% FBS) or subjected to serum-free conditions in RPMI medium without phenol red during 48 h. After serum starvation, cells were incubated with 10 nM R5020 or vehicle (ethanol; EtOH) for the indicated times.

### ChIP assays

ChIP assays were performed as described [71] using anti-CHD8 (A301-224A) from Bethyl Laboratories or home-made rabbit anti-CHD8 [16], anti-RNAPII (N-20) (sc-899), anti-PR (H-190) (sc-7208), anti-BAF155 (R-18) (sc-9746) and anti-FOXA1 (H-120) (sc-22841) from Santa Cruz Biotechnology, and anti-H3K27Ac (ab4729) from Abcam. Chromatin was sonicated to an average fragment size of 400 to 500 bp using the Diagenode Bioruptor. Rabbit IgG (Sigma) was used as a control for non-specific interactions. Input was prepared with 10% of the chromatin material used for immunoprecipitation. Input material was diluted 1:10 before PCR amplification. Quantification of immunoprecipitated DNA was performed by real-time PCR (qPCR) with the Applied Biosystems 7500 FAST real-time PCR system, using Applied Biosystems Power SYBR green master mix. Sample quantifications by qPCR were performed in triplicate. Sequences of all oligonucleotides are available upon request. Data are the average of at least three independent experiments.

### ChIP-seq

ChIP was performed as described above using anti-CHD8 (A301-224A, Bethyl Laboratories). ChIP-DNA was purified and subjected to deep sequencing using the Solexa Genome Analyzer (Illumina). The sequence reads were aligned to the human genome reference (assembly hg19). ChIP-seq peak calling, genomic annotation of peaks and comparison between ChIP-seq and ENCODE datasets were performed using ChIPseeqer (v.2.1) [72]. An empirical approach was followed to estimate the FDR, which involves using control data set as ChIP-seq data and the ChIP-seq data as the pseudo-control data and running peak detection. The FDR is defined as the ratio of the number of peaks detected in this pseudo-control analysis, to the number of peaks detected in the real ChIP-seq experiment. Motif analysis was performed using FIRE algorithm [73], included in the ChIPseeqer framework, MEME suite [74], Weeder PScan [75] and TRANSFAC database [76]. CHD8 ChIP-seq data are available from the GEO database (accession number GSE49134). RNAPII, H3K4me3 and PR ChIP-seq data in T47D were previously reported [25]. GEO accession number for FOXA1 and CTCF ChIP-seq data are GMS803409, GSM803348.

### RNAi experiments

All siRNAs were transfected using Oligofectamine (Invitrogen) according to the manufacturer’s instructions, with following siRNA sequences: for siCHD8, 5′-GAGCAAGCUCAACACCAUC-3′; siFOXA1, 5′-GAGAGAAAAAAUCAACAGC-3′; siCt, 5′-CGUACGCGGAAUACUUCGA-3′; siBRG1, 5′-GCGACUCACUGACGGAGAA-3′; and siBRM, 5′-GAAAGGAGGUGCUAAGACA-3′. After transfection, medium was replaced by serum-free fresh medium without phenol red. After 48 h in serum-free conditions, cells were treated with 10 nM R5020 or vehicle (EtOH) for the indicated times. The down-regulation of CHD8, FOXA1, BRG1 and BRM was confirmed by RT-PCR and Western blotting, respectively.

### Microarray expression analysis

T47D-MTVL cells were grown in serum-free conditions, as explained previously, during 48 h and treated with 10 nM R5020 or vehicle (EtOH) during 6 h. Total RNA was isolated in triplicate from cells using RNeasy Mini Kit (Qiagen). Purity and quality of isolated RNA were assessed by RNA 6000 Nano assay on a 2100 Bioanalyzer (Agilent Technologies, Santa 6 Clara, CA). RNA (100 ng) was used for production of end-labelled biotinylated ssDNA. Labelled ssDNA was hybridized to the GeneChip human Gene 1.0 ST array oligonucleotide microarray (Affymetrix, Santa Clara, CA) according to manufacturer’s recommendations. The arrays were scanned using the GeneChip Scanner 3000 7G (Affymetrix), and raw data were extracted from the scanned images and analyzed with the Affymetrix GeneChip Command Console Software (Affymetrix). The raw array data were pre-processed and normalized using the Robust Multichip Average (RMA) method [77]. Data were further processed using oneChannelGUI [78]. The log2 intensities for each probe were used for further analysis. Genes where considered as hormone induced when change of gene expression was >1.5 (linear fold change) and p-value < 0.01. Gene expression was considered as CHD8-dependent when [hormone-dependent change in siCHD8]/[hormone-dependent change in siCt] was higher than 1.20 or lower than 0.8. Microarray data are available from the GEO database (accession number GSE62257).

### DNase I assay

DNase I assay was performed as previously described [40]. Briefly, 2 μg of crosslinked chromatin were treated with 0.5, 1, and 2 units of DNase I (Roche) for 3 min at 37°C. Control samples were incubated in the absence of DNaseI. Reactions were stopped by adding EDTA and the crosslinking was reversed by incubating the samples at 65°C. DNA was then isolated, quantified and used as template for qPCR reactions using specific primers.

### RNA extraction and RT-PCR

Total RNA was prepared by using the RNeasy Kit (Qiagen), as described in the manufacturer’s instructions; note that the step of DNase I digestion was included to avoid potential DNA contamination. cDNA was generated from 800 ng of total RNA (for progesterone-dependent mRNA induction analysis) using Superscript First Strand Synthesis System (Invitrogen). For progesterone-dependent eRNA induction analysis 2 μg of total RNA was used. In the case of *FKBP5e* and *HSD11B2e* eRNA determination, strand-specific oligonucleotides were used for RT, in order to avoid expression form close promoters. cDNA (2 μl) was used as a template for qPCR. Gene products were quantified by real-time PCR with the Applied Biosystems 7500 FAST real-time PCR system, using Applied Biosystems Power SYBR green master mix. Sequences of all oligonucleotides are available upon request. Values were normalized to the expression of the 28S housekeeping gene. Each experiment was performed at least in duplicate, and qPCR quantifications were performed in triplicate.

### Co-immunoprecipitation assays and western blot

Co-immunoprecipitations were performed as described in [17] using the anti-CHD8 antibody (A301-224A) from Bethyl Laboratories. Rabbit or mouse purified IgG (Sigma-Aldrich) were used as a control. 3% Input and precipitated proteins were separated by SDS/PAGE, and visualized by Western blotting with the indicated antibodies using ECL Plus (GE Healthcare), according to the manufacturer’s instructions. Antibodies used for western blotting were: anti-BRG1 (H88, sc-10768), anti-BAF155 (R-18, sc-9746), anti-BAF170 (E-6, sc-17838), anti-PR (H-190) (sc-7208) and anti-FOXA1 (H-120) (sc-22841), and anti-hSNF5 (C20, sc-16189) from Santa Cruz Biotechnology; anti-CHD8 (A301-224A) and anti-BAF180 (A301-590A) from Bethyl; anti-BAF250 (04–080) from Millipore; α-tubulin antibody (DM1A, T9026) from Sigma Aldrich and anti-BRM (ab15597) from Abcam.

## Supporting Information

S1 Fig CHD8 binds gene promoters in proliferating T47D-MTVL cells. (A) ChIP-qPCR analysis of CHD8 binding to *CCND1*, *HDS11B2* and *CCNE2* promoters using a home-made anti-CHD8 antibody [16]. Pre-immune serum (PreI) was used as negative control. (B) CHD8 ChIP signal was reduced upon knockdown of CHD8 by siRNA. ChIP-qPCR analysis of CHD8 using anti-CHD8 antibody (A301-224A, Bethyl Laboratories) at the indicated promoters, in T47D-MTVL cells transfected with control siRNA (siCt) or siRNA against CHD8 (siCHD8).
(PDF)

S2 Fig Analysis of CHD8 binding sites in proliferating T47D-MTVL cells. (A) Gene ontology functional categories of CHD8 target genes in proliferating T47D-MTVL cells, analyzed by DAVID [79]. The numbers at the right represent the statistical enrichment *(P* value) and the bars represent the percentage of CHD8 target genes within a functional category. (B) The top transcription factor binding motifs of CHD8 bound promoters in proliferating T47D-MTVL cells, analyzed using Weeder PScan [75] and TRANSFAC database [76]. The associated logo with the matrix and the statistical enrichment (*P*-value) are also shown.
(PDF)

S3 Fig CHD8 enrichment at TSS after hormone treatment. CHD8 occupancy after 5 (red) or 45 (green) min of R5020 treatment, plotted as the normalized tag density around TSS.
(PDF)

S4 Fig CHD8 enrichment at H3K4me1 sites after hormone treatment. CHD8 occupancy after 5 (red) or 45 (green) min of R5020 treatment, plotted as the normalized tag density around the centre of H3K4me1 enriched regions.
(PDF)

S5 Fig Analysis of overlapping between CHD8 and CTCF sites. (A) Venn-diagram showing overlap between CHD8 and CTCF peaks in proliferating T47D-MTVL cells. (B) Distribution of CHD8-CTCF co-occupied sites, relative to known RefSeq genes. Promoters: ± 2 kb around transcription start site (TSS); Downstream extremities: ± 2 kb around transcription end site; Exons: exonic regions; Introns: intronic regions; Intergenic > 2 kb away from RefSeq TSS. (C) Venn-diagram showing overlap between CHD8 and CTCF peaks in T47D-MTVL cells upon hormone induction with R5020.
(PDF)

S6 Fig Effect of CHD8 depletion in progestin-dependent expression. T47D-MTVL cells were transfected with a control siRNA or siCHD8-2 specifically targeting CHD8. Forty-eight hours after transfection cells were stimulated with progestin (R5020) or vehicle (EtOH) for 45 min. Expression of the following genes: *HSD11B2*, *MMTV-Luc*, *DUSP1*, *FKBP5*, *NFE2L3* and *IL6ST* was monitored by RT-qPCR. Level of *CHD8* expression was determined as control of silencing.
(PDF)

S7 Fig SWI/SNF complexes interact with CHD8. (A) SWI/SNF subunits co-immunoprecipitate with CHD8 both in the presence and in the absence of progestin. Extract from T47D-MTVL cells treated with R5020 (+) or vehicle (-) for 45 min were subjected to immunoprecipitation using anti-CHD8 antibody. Precipitated proteins were then revealed by western blotting using the indicated antibodies. (B) RT-qPCR analysis of *BRG1* and *BRM* expression upon transfection of T47D-MTVL cells with control siRNA (siCt) or a pool of siRNAs against *BRM* and *BRG1* (siBRG1+siBRM). After 48 hours, cells were stimulated during 45 min with progestin (R5020) or vehicle (EtOH). (C) Western blot analysis of BRG1 and BRM expression upon transfection of T47D-MTVL cells with control siRNA (siCt) or siRNAs against BRG1 and BRM (siBRG1 siBRM).
(PDF)

## References

[1] Lenhard B, Sandelin A, Carninci P (2012) Metazoan promoters: emerging characteristics and insights into transcriptional regulation. Nat Rev Genet 13: 233–245. 10.1038/nrg3163 22392219

[2] Calo E, Wysocka J (2013) Modification of enhancer chromatin: what, how, and why? Mol Cell 49: 825–837. 10.1016/j.molcel.2013.01.038 23473601 PMC3857148

[3] Maston GA, Landt SG, Snyder M, Green MR (2012) Characterization of enhancer function from genome-wide analyses. Annu Rev Genomics Hum Genet 13: 29–57. 10.1146/annurev-genom-090711-163723 22703170

[4] Voss TC, Hager GL (2014) Dynamic regulation of transcriptional states by chromatin and transcription factors. Nat Rev Genet 15: 69–81. 10.1038/nrg3623 24342920 PMC6322398

[5] Maston GA, Evans SK, Green MR (2006) Transcriptional regulatory elements in the human genome. Annu Rev Genomics Hum Genet 7: 29–59. 16719718 10.1146/annurev.genom.7.080505.115623

[6] Hargreaves DC, Crabtree GR (2011) ATP-dependent chromatin remodeling: genetics, genomics and mechanisms. Cell Res 21: 396–420. 10.1038/cr.2011.32 21358755 PMC3110148

[7] Narlikar GJ, Sundaramoorthy R, Owen-Hughes T (2013) Mechanisms and Functions of ATP-Dependent Chromatin-Remodeling Enzymes. Cell 154: 490–503. 10.1016/j.cell.2013.07.011 23911317 PMC3781322

[8] Marfella CG, Imbalzano AN (2007) The Chd family of chromatin remodelers. Mutat Res 618: 30–40. 17350655 10.1016/j.mrfmmm.2006.07.012 PMC1899158

[9] Thompson BA, Tremblay V, Lin G, Bochar DA (2008) CHD8 is an ATP-dependent chromatin remodeling factor that regulates beta-catenin target genes. Mol Cell Biol 28: 3894–3904. 10.1128/MCB.00322-08 18378692 PMC2423111

[10] Nishiyama M, Nakayama K, Tsunematsu R, Tsukiyama T, Kikuchi A, et al (2004) Early embryonic death in mice lacking the beta-catenin-binding protein Duplin. Mol Cell Biol 24: 8386–8394. 15367660 10.1128/MCB.24.19.8386-8394.2004 PMC516734

[11] Ishihara K, Oshimura M, Nakao M (2006) CTCF-dependent chromatin insulator is linked to epigenetic remodeling. Mol Cell 23: 733–742. 16949368 10.1016/j.molcel.2006.08.008

[12] Sakamoto I, Kishida S, Fukui A, Kishida M, Yamamoto H, et al (2000) A novel beta-catenin-binding protein inhibits beta-catenin-dependent Tcf activation and axis formation. J Biol Chem 275: 32871–32878. 10921920 10.1074/jbc.M004089200

[13] Nishiyama M, Oshikawa K, Tsukada Y, Nakagawa T, Iemura S, et al (2009) CHD8 suppresses p53-mediated apoptosis through histone H1 recruitment during early embryogenesis. Nat Cell Biol 11: 172–182. 10.1038/ncb1831 19151705 PMC3132516

[14] Nishiyama M, Skoultchi AI, Nakayama KI (2012) Histone H1 recruitment by CHD8 is essential for suppression of the Wnt-beta-catenin signaling pathway. Mol Cell Biol 32: 501–512. 10.1128/MCB.06409-11 22083958 PMC3255766

[15] Fang M, Ou J, Hutchinson L, Green MR (2014) The BRAF oncoprotein functions through the transcriptional repressor MAFG to mediate the CpG Island Methylator phenotype. Mol Cell 55: 904–915. 10.1016/j.molcel.2014.08.010 25219500 PMC4170521

[16] Rodriguez-Paredes M, Ceballos-Chavez M, Esteller M, Garcia-Dominguez M, Reyes JC (2009) The chromatin remodeling factor CHD8 interacts with elongating RNA polymerase II and controls expression of the cyclin E2 gene. Nucleic Acids Res 37: 2449–2460. 10.1093/nar/gkp101 19255092 PMC2677868

[17] Subtil-Rodriguez A, Vazquez-Chavez E, Ceballos-Chavez M, Rodriguez-Paredes M, Martin-Subero JI, et al (2014) The chromatin remodeller CHD8 is required for E2F-dependent transcription activation of S-phase genes. Nucleic Acids Res 42: 2185–2196. 10.1093/nar/gkt1161 24265227 PMC3936757

[18] Caldon CE, Sergio CM, Schutte J, Boersma MN, Sutherland RL, et al (2009) Estrogen regulation of cyclin E2 requires cyclin D1 but not c-Myc. Mol Cell Biol 29: 4623–4639. 10.1128/MCB.00269-09 19564413 PMC2725719

[19] Menon T, Yates JA, Bochar DA (2010) Regulation of androgen-responsive transcription by the chromatin remodeling factor CHD8. Mol Endocrinol 24: 1165–1174. 10.1210/me.2009-0421 20308527 PMC2875808

[20] Beato M, Vicent GP (2012) Impact of chromatin structure and dynamics on PR signaling. The initial steps in hormonal gene regulation. Mol Cell Endocrinol 357: 37–42. 10.1016/j.mce.2011.09.004 21945605

[21] Vicent GP, Nacht AS, Font-Mateu J, Castellano G, Gaveglia L, et al (2011) Four enzymes cooperate to displace histone H1 during the first minute of hormonal gene activation. Genes Dev 25: 845–862. 10.1101/gad.621811 21447625 PMC3078709

[22] Vicent GP, Zaurin R, Nacht AS, Li A, Font-Mateu J, et al (2009) Two chromatin remodeling activities cooperate during activation of hormone responsive promoters. PLoS Genet 5: e1000567 10.1371/journal.pgen.1000567 19609353 PMC2704372

[23] Vicent GP, Ballare C, Nacht AS, Clausell J, Subtil-Rodriguez A, et al (2006) Induction of progesterone target genes requires activation of Erk and Msk kinases and phosphorylation of histone H3. Mol Cell 24: 367–381. 17081988 10.1016/j.molcel.2006.10.011

[24] Pedram A, Razandi M, Sainson RC, Kim JK, Hughes CC, et al (2007) A conserved mechanism for steroid receptor translocation to the plasma membrane. J Biol Chem 282: 22278–22288. 17535799 10.1074/jbc.M611877200

[25] Ballaré C, Castellano G, Gaveglia L, Althammer S, Gonzalez-Vallinas J, et al (2013) Nucleosome-driven transcription factor binding and gene regulation. Mol Cell 49: 67–79. 10.1016/j.molcel.2012.10.019 23177737

[26] Yin P, Roqueiro D, Huang L, Owen JK, Xie A, et al (2012) Genome-wide progesterone receptor binding: cell type-specific and shared mechanisms in T47D breast cancer cells and primary leiomyoma cells. PLoS One 7: e29021 10.1371/journal.pone.0029021 22272226 PMC3260146

[27] Clarke CL, Graham JD (2012) Non-overlapping progesterone receptor cistromes contribute to cell-specific transcriptional outcomes. PLoS One 7: e35859 10.1371/journal.pone.0035859 22545144 PMC3335806

[28] Truss M, Bartsch J, Schelbert A, Hache RJ, Beato M (1995) Hormone induces binding of receptors and transcription factors to a rearranged nucleosome on the MMTV promoter in vivo. Embo J 14: 1737–1751. 7737125 10.1002/j.1460-2075.1995.tb07163.x PMC398267

[29] Visel A, Blow MJ, Li Z, Zhang T, Akiyama JA, et al (2009) ChIP-seq accurately predicts tissue-specific activity of enhancers. Nature 457: 854–858. 10.1038/nature07730 19212405 PMC2745234

[30] Heintzman ND, Hon GC, Hawkins RD, Kheradpour P, Stark A, et al (2009) Histone modifications at human enhancers reflect global cell-type-specific gene expression. Nature 459: 108–112. 10.1038/nature07829 19295514 PMC2910248

[31] Cuddapah S, Jothi R, Schones DE, Roh TY, Cui K, et al (2009) Global analysis of the insulator binding protein CTCF in chromatin barrier regions reveals demarcation of active and repressive domains. Genome Res 19: 24–32. 10.1101/gr.082800.108 19056695 PMC2612964

[32] Sartorius CA, Groshong SD, Miller LA, Powell RL, Tung L, et al (1994) New T47D breast cancer cell lines for the independent study of progesterone B- and A-receptors: only antiprogestin-occupied B-receptors are switched to transcriptional agonists by cAMP. Cancer Res 54: 3868–3877. 8033109

[33] Zaret KS, Carroll JS (2011) Pioneer transcription factors: establishing competence for gene expression. Genes Dev 25: 2227–2241. 10.1101/gad.176826.111 22056668 PMC3219227

[34] Lupien M, Eeckhoute J, Meyer CA, Wang Q, Zhang Y, et al (2008) FoxA1 translates epigenetic signatures into enhancer-driven lineage-specific transcription. Cell 132: 958–970. 10.1016/j.cell.2008.01.018 18358809 PMC2323438

[35] Hurtado A, Holmes KA, Ross-Innes CS, Schmidt D, Carroll JS (2011) FOXA1 is a key determinant of estrogen receptor function and endocrine response. Nat Genet 43: 27–33. 10.1038/ng.730 21151129 PMC3024537

[36] Creyghton MP, Cheng AW, Welstead GG, Kooistra T, Carey BW, et al (2010) Histone H3K27ac separates active from poised enhancers and predicts developmental state. Proc Natl Acad Sci U S A 107: 21931–21936. 10.1073/pnas.1016071107 21106759 PMC3003124

[37] Rada-Iglesias A, Bajpai R, Prescott S, Brugmann SA, Swigut T, et al (2012) Epigenomic annotation of enhancers predicts transcriptional regulators of human neural crest. Cell Stem Cell 11: 633–648. 10.1016/j.stem.2012.07.006 22981823 PMC3751405

[38] Rada-Iglesias A, Bajpai R, Swigut T, Brugmann SA, Flynn RA, et al (2011) A unique chromatin signature uncovers early developmental enhancers in humans. Nature 470: 279–283. 10.1038/nature09692 21160473 PMC4445674

[39] Tie F, Banerjee R, Stratton CA, Prasad-Sinha J, Stepanik V, et al (2009) CBP-mediated acetylation of histone H3 lysine 27 antagonizes Drosophila Polycomb silencing. Development 136: 3131–3141. 10.1242/dev.037127 19700617 PMC2730368

[40] Di Stefano B, Sardina JL, van Oevelen C, Collombet S, Kallin EM, et al (2014) C/EBPalpha poises B cells for rapid reprogramming into induced pluripotent stem cells. Nature 506: 235–239. 10.1038/nature12885 24336202

[41] De Santa F, Barozzi I, Mietton F, Ghisletti S, Polletti S, et al (2010) A large fraction of extragenic RNA pol II transcription sites overlap enhancers. PLoS Biol 8: e1000384 10.1371/journal.pbio.1000384 20485488 PMC2867938

[42] Djebali S, Davis CA, Merkel A, Dobin A, Lassmann T, et al (2012) Landscape of transcription in human cells. Nature 489: 101–108. 10.1038/nature11233 22955620 PMC3684276

[43] Kim TK, Hemberg M, Gray JM, Costa AM, Bear DM, et al (2010) Widespread transcription at neuronal activity-regulated enhancers. Nature 465: 182–187. 10.1038/nature09033 20393465 PMC3020079

[44] Wang D, Garcia-Bassets I, Benner C, Li W, Su X, et al (2011) Reprogramming transcription by distinct classes of enhancers functionally defined by eRNA. Nature 474: 390–394. 10.1038/nature10006 21572438 PMC3117022

[45] Hah N, Murakami S, Nagari A, Danko CG, Kraus WL (2013) Enhancer transcripts mark active estrogen receptor binding sites. Genome Res 23: 1210–1223. 10.1101/gr.152306.112 23636943 PMC3730096

[46] Yuan CC, Zhao X, Florens L, Swanson SK, Washburn MP, et al (2007) CHD8 associates with human Staf and contributes to efficient U6 RNA polymerase III transcription. Mol Cell Biol 27: 8729–8738. 17938208 10.1128/MCB.00846-07 PMC2169411

[47] Sugathan A, Biagioli M, Golzio C, Erdin S, Blumenthal I, et al (2014) CHD8 regulates neurodevelopmental pathways associated with autism spectrum disorder in neural progenitors. Proc Natl Acad Sci U S A 111: E4468–4477. 10.1073/pnas.1405266111 25294932 PMC4210312

[48] Batsukh T, Pieper L, Koszucka AM, von Velsen N, Hoyer-Fender S, et al (2010) CHD8 interacts with CHD7, a protein which is mutated in CHARGE syndrome. Hum Mol Genet 19: 2858–2866. 10.1093/hmg/ddq189 20453063

[49] Schnetz MP, Bartels CF, Shastri K, Balasubramanian D, Zentner GE, et al (2009) Genomic distribution of CHD7 on chromatin tracks H3K4 methylation patterns. Genome Res 19: 590–601. 10.1101/gr.086983.108 19251738 PMC2665778

[50] Schnetz MP, Handoko L, Akhtar-Zaidi B, Bartels CF, Pereira CF, et al (2010) CHD7 targets active gene enhancer elements to modulate ES cell-specific gene expression. PLoS Genet 6: e1001023 10.1371/journal.pgen.1001023 20657823 PMC2904778

[51] Melgar MF, Collins FS, Sethupathy P (2011) Discovery of active enhancers through bidirectional expression of short transcripts. Genome Biol 12: R113 10.1186/gb-2011-12-11-r113 22082242 PMC3334599

[52] Degner SC, Verma-Gaur J, Wong TP, Bossen C, Iverson GM, et al (2011) CCCTC-binding factor (CTCF) and cohesin influence the genomic architecture of the Igh locus and antisense transcription in pro-B cells. Proc Natl Acad Sci U S A 108: 9566–9571. 10.1073/pnas.1019391108 21606361 PMC3111298

[53] Dawson MA, Prinjha RK, Dittmann A, Giotopoulos G, Bantscheff M, et al (2011) Inhibition of BET recruitment to chromatin as an effective treatment for MLL-fusion leukaemia. Nature 478: 529–533. 10.1038/nature10509 21964340 PMC3679520

[54] Ho L, Jothi R, Ronan JL, Cui K, Zhao K, et al (2009) An embryonic stem cell chromatin remodeling complex, esBAF, is an essential component of the core pluripotency transcriptional network. Proc Natl Acad Sci U S A 106: 5187–5191. 10.1073/pnas.0812888106 19279218 PMC2654397

[55] Bajpai R, Chen DA, Rada-Iglesias A, Zhang J, Xiong Y, et al (2010) CHD7 cooperates with PBAF to control multipotent neural crest formation. Nature 463: 958–962. 10.1038/nature08733 20130577 PMC2890258

[56] Nagl NG Jr., Wang X, Patsialou A, Van Scoy M, Moran E (2007) Distinct mammalian SWI/SNF chromatin remodeling complexes with opposing roles in cell-cycle control. Embo J 26: 752–763. 17255939 10.1038/sj.emboj.7601541 PMC1794396

[57] Heintzman ND, Stuart RK, Hon G, Fu Y, Ching CW, et al (2007) Distinct and predictive chromatin signatures of transcriptional promoters and enhancers in the human genome. Nat Genet 39: 311–318. 17277777 10.1038/ng1966

[58] Kaikkonen MU, Spann NJ, Heinz S, Romanoski CE, Allison KA, et al (2013) Remodeling of the enhancer landscape during macrophage activation is coupled to enhancer transcription. Mol Cell 51: 310–325. 10.1016/j.molcel.2013.07.010 23932714 PMC3779836

[59] Pekowska A, Benoukraf T, Zacarias-Cabeza J, Belhocine M, Koch F, et al (2011) H3K4 tri-methylation provides an epigenetic signature of active enhancers. Embo J 30: 4198–4210. 10.1038/emboj.2011.295 21847099 PMC3199384

[60] Mousavi K, Zare H, Dell'orso S, Grontved L, Gutierrez-Cruz G, et al (2013) eRNAs Promote Transcription by Establishing Chromatin Accessibility at Defined Genomic Loci. Mol Cell 51: 606–617. 10.1016/j.molcel.2013.07.022 23993744 PMC3786356

[61] Melo CA, Drost J, Wijchers PJ, van de Werken H, de Wit E, et al (2013) eRNAs are required for p53-dependent enhancer activity and gene transcription. Mol Cell 49: 524–535. 10.1016/j.molcel.2012.11.021 23273978

[62] Li W, Notani D, Ma Q, Tanasa B, Nunez E, et al (2013) Functional roles of enhancer RNAs for oestrogen-dependent transcriptional activation. Nature 498: 516–520. 10.1038/nature12210 23728302 PMC3718886

[63] Lam MT, Cho H, Lesch HP, Gosselin D, Heinz S, et al (2013) Rev-Erbs repress macrophage gene expression by inhibiting enhancer-directed transcription. Nature 498: 511–515. 10.1038/nature12209 23728303 PMC3839578

[64] Lai F, Orom UA, Cesaroni M, Beringer M, Taatjes DJ, et al (2013) Activating RNAs associate with Mediator to enhance chromatin architecture and transcription. Nature 494: 497–501. 10.1038/nature11884 23417068 PMC4109059

[65] Srinivasan S, Armstrong JA, Deuring R, Dahlsveen IK, McNeill H, et al (2005) The Drosophila trithorax group protein Kismet facilitates an early step in transcriptional elongation by RNA Polymerase II. Development 132: 1623–1635. 15728673 10.1242/dev.01713

[66] Garel A, Axel R (1976) Selective digestion of transcriptionally active ovalbumin genes from oviduct nuclei. Proc Natl Acad Sci U S A 73: 3966–3970. 1069279 10.1073/pnas.73.11.3966 PMC431285

[67] Weintraub H, Groudine M (1976) Chromosomal subunits in active genes have an altered conformation. Science 193: 848–856. 948749 10.1126/science.948749

[68] Marom R, Shur I, Hager GL, Benayahu D (2006) Expression and regulation of CReMM, a chromodomain helicase-DNA-binding (CHD), in marrow stroma derived osteoprogenitors. J Cell Physiol 207: 628–635. 16523501 10.1002/jcp.20611

[69] Surapureddi S, Viswakarma N, Yu S, Guo D, Rao MS, et al (2006) PRIC320, a transcription coactivator, isolated from peroxisome proliferator-binding protein complex. Biochem Biophys Res Commun 343: 535–543. 16554032 10.1016/j.bbrc.2006.02.160

[70] Jacobsen BM, Richer JK, Schittone SA, Horwitz KB (2002) New human breast cancer cells to study progesterone receptor isoform ratio effects and ligand-independent gene regulation. J Biol Chem 277: 27793–27800. 12021276 10.1074/jbc.M202584200

[71] Strutt H, Paro R (1999) Mapping DNA target sites of chromatin proteins in vivo by formaldehyde crosslinking. Methods Mol Biol 119: 455–467. 10804532 10.1385/1-59259-681-9:455

[72] Giannopoulou EG, Elemento O (2011) An integrated ChIP-seq analysis platform with customizable workflows. BMC Bioinformatics 12: 277 10.1186/1471-2105-12-277 21736739 PMC3145611

[73] Elemento O, Slonim N, Tavazoie S (2007) A universal framework for regulatory element discovery across all genomes and data types. Mol Cell 28: 337–350. 17964271 10.1016/j.molcel.2007.09.027 PMC2900317

[74] Bailey TL, Boden M, Buske FA, Frith M, Grant CE, et al (2009) MEME SUITE: tools for motif discovery and searching. Nucleic Acids Res 37: W202–208. 10.1093/nar/gkp335 19458158 PMC2703892

[75] Pavesi G, Mereghetti P, Mauri G, Pesole G (2004) Weeder Web: discovery of transcription factor binding sites in a set of sequences from co-regulated genes. Nucleic Acids Res 32: W199–203. 15215380 10.1093/nar/gkh465 PMC441603

[76] Matys V, Kel-Margoulis OV, Fricke E, Liebich I, Land S, et al (2006) TRANSFAC and its module TRANSCompel: transcriptional gene regulation in eukaryotes. Nucleic Acids Res 34: D108–110. 16381825 10.1093/nar/gkj143 PMC1347505

[77] Irizarry RA, Bolstad BM, Collin F, Cope LM, Hobbs B, et al (2003) Summaries of Affymetrix GeneChip probe level data. Nucleic Acids Res 31: e15 12582260 10.1093/nar/gng015 PMC150247

[78] Sanges R, Cordero F, Calogero RA (2007) oneChannelGUI: a graphical interface to Bioconductor tools, designed for life scientists who are not familiar with R language. Bioinformatics 23: 3406–3408. 17875544 10.1093/bioinformatics/btm469

[79] Huang da W, Sherman BT, Lempicki RA (2009) Systematic and integrative analysis of large gene lists using DAVID bioinformatics resources. Nat Protoc 4: 44–57. 10.1038/nprot.2008.211 19131956

