# Supplementary figures and images for: The Chromatin Remodeler CHD8 Is Required for Activation of Progesterone Receptor-Dependent Enhancers
Source: PLoS Genet. 2015 Apr 20;11(4):e1005174. doi: 10.1371/journal.pgen.1005174 (PMC4403880; doi:10.1371/journal.pgen.1005174)

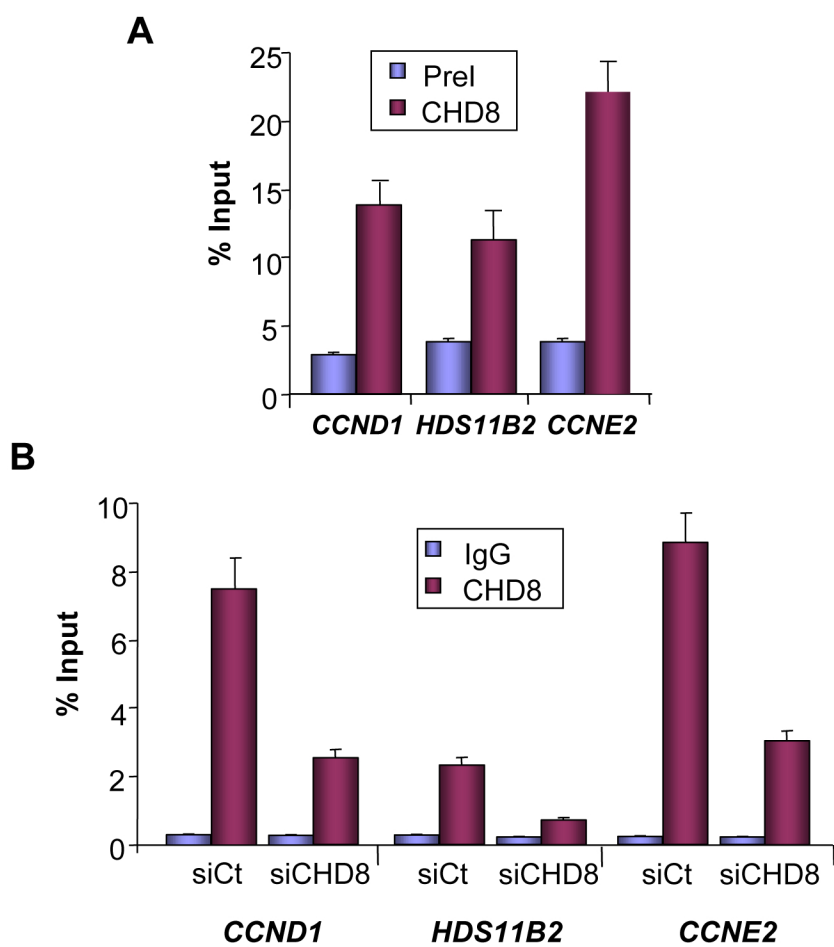

**Fig. S1**

Supplement: S1 Fig — (A) ChIP-qPCR analysis of CHD8 binding to CCND1, HDS11B2 and CCNE2 promoters using a home-made anti-CHD8 antibody [16]. Pre-immune serum (PreI) was used as negative control. (B) CHD8 ChIP signal was reduced upon knockdown of CHD8 by siRNA. ChIP-qPCR analysis of CHD8 using anti-CHD8 antibody (A301-224A, Bethyl Laboratories) at the indicated promoters, in T47D-MTVL cells transfected with control siRNA (siCt) or siRNA against CHD8 (siCHD8). (PDF) [file pgen.1005174.s001.pdf]

**A**

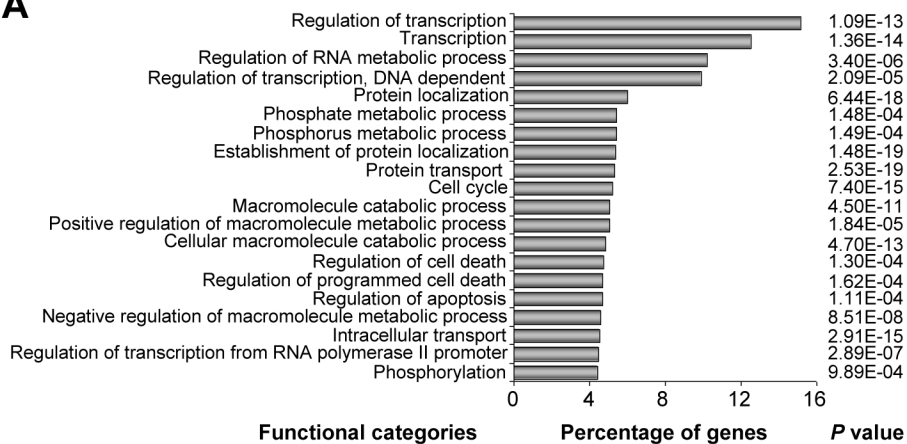

**B**

| Transcription Factor | Matrix Logo | P value   |
|----------------------|-------------|-----------|
| E2F                  |             | 3.65E-135 |
| Elk-1                |             | 2.24E-122 |
| AP-2                 |             | 4.74E-100 |
| E2F                  |             | 5.21E-82  |
| Sp1                  |             | 4.18E-80  |
| AP-2alpha            |             | 2.35E-67  |

**Fig. S2**

Supplement: S2 Fig — (A) Gene ontology functional categories of CHD8 target genes in proliferating T47D-MTVL cells, analyzed by DAVID [79]. The numbers at the right represent the statistical enrichment (P value) and the bars represent the percentage of CHD8 target genes within a functional category. (B) The top transcription factor binding motifs of CHD8 bound promoters in proliferating T47D-MTVL cells, analyzed using Weeder PScan [75] and TRANSFAC database [76]. The associated logo with the matrix and the statistical enrichment (P-value) are also shown. (PDF) [file pgen.1005174.s002.pdf]

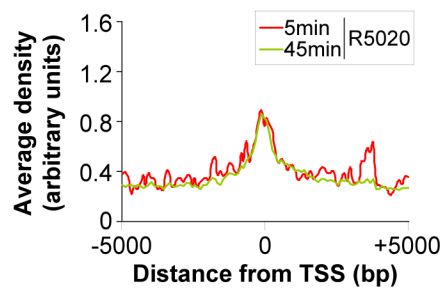

**Fig. S3**

Supplement: S3 Fig — CHD8 occupancy after 5 (red) or 45 (green) min of R5020 treatment, plotted as the normalized tag density around TSS. (PDF) [file pgen.1005174.s003.pdf]

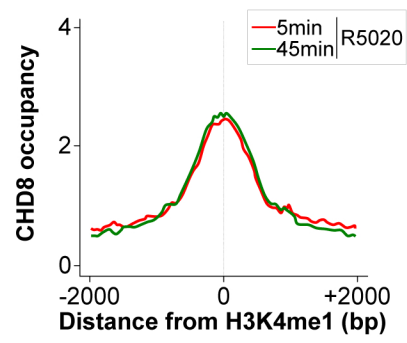

**Fig. S4**

Supplement: S4 Fig — CHD8 occupancy after 5 (red) or 45 (green) min of R5020 treatment, plotted as the normalized tag density around the centre of H3K4me1 enriched regions. (PDF) [file pgen.1005174.s004.pdf]

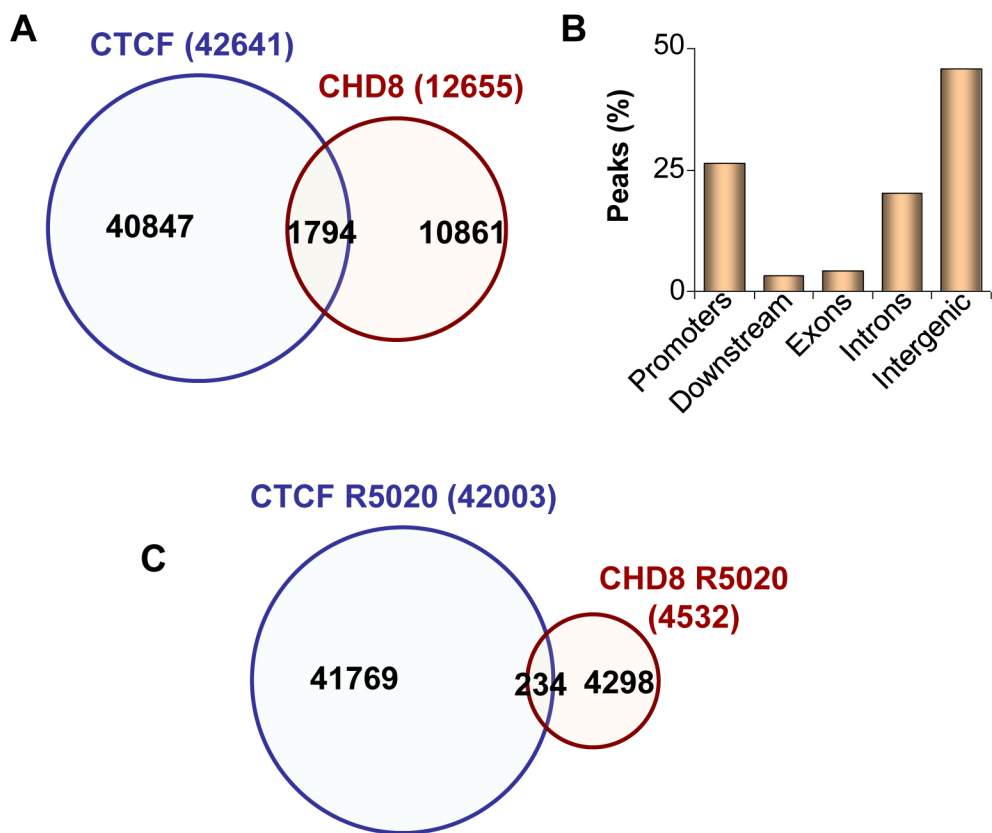

**Fig. S5**

Supplement: S5 Fig — (A) Venn-diagram showing overlap between CHD8 and CTCF peaks in proliferating T47D-MTVL cells. (B) Distribution of CHD8-CTCF co-occupied sites, relative to known RefSeq genes. Promoters: ± 2 kb around transcription start site (TSS); Downstream extremities: ± 2 kb around transcription end site; Exons: exonic regions; Introns: intronic regions; Intergenic > 2 kb away from RefSeq TSS. (C) Venn-diagram showing overlap between CHD8 and CTCF peaks in T47D-MTVL cells upon hormone induction with R5020. (PDF) [file pgen.1005174.s005.pdf]

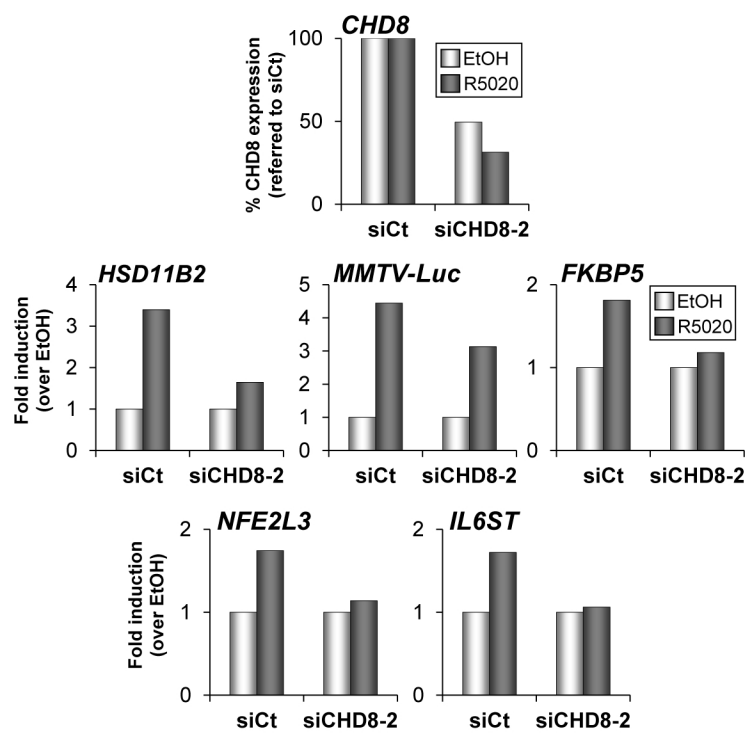

Fig. S6

Supplement: S6 Fig — T47D-MTVL cells were transfected with a control siRNA or siCHD8-2 specifically targeting CHD8. Forty-eight hours after transfection cells were stimulated with progestin (R5020) or vehicle (EtOH) for 45 min. Expression of the following genes: HSD11B2, MMTV-Luc, DUSP1, FKBP5, NFE2L3 and IL6ST was monitored by RT-qPCR. Level of CHD8 expression was determined as control of silencing. (PDF) [file pgen.1005174.s006.pdf]

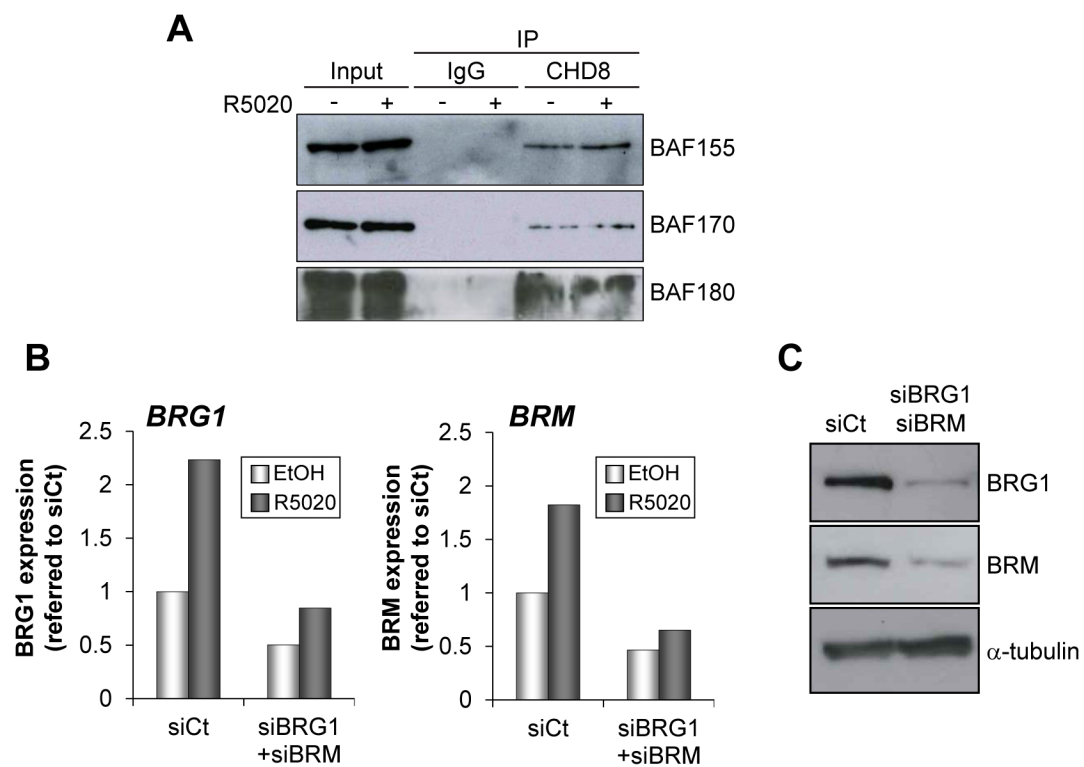

**Fig. S7**

Supplement: S7 Fig — (A) SWI/SNF subunits co-immunoprecipitate with CHD8 both in the presence and in the absence of progestin. Extract from T47D-MTVL cells treated with R5020 (+) or vehicle (-) for 45 min were subjected to immunoprecipitation using anti-CHD8 antibody. Precipitated proteins were then revealed by western blotting using the indicated antibodies. (B) RT-qPCR analysis of BRG1 and BRM expression upon transfection of T47D-MTVL cells with control siRNA (siCt) or a pool of siRNAs against BRM and BRG1 (siBRG1+siBRM). After 48 hours, cells were stimulated during 45 min with progestin (R5020) or vehicle (EtOH). (C) Western blot analysis of BRG1 and BRM expression upon transfection of T47D-MTVL cells with control siRNA (siCt) or siRNAs against BRG1 and BRM (siBRG1 siBRM). (PDF) [file pgen.1005174.s007.pdf]
